# Cytoplasmic SHMT2 drives the progression and metastasis of colorectal cancer by inhibiting β-catenin degradation

**DOI:** 10.7150/thno.48699

**Published:** 2021-01-01

**Authors:** Chunqi Liu, Liang Wang, Xiaocong Liu, Yuping Tan, Lei Tao, Yuzhou Xiao, Pengchi Deng, Huijuan Wang, Qianyi Deng, Yiyun Lin, Hui Jie, Huaqin Zhang, Jing Zhang, Yong Peng, Hu Zhang, Zongguang Zhou, Qingxiang Sun, Xiaobo Cen, Yinglan Zhao

**Affiliations:** 1 State Key Laboratory of Biotherapy and Cancer Center, West China Hospital, West China Medical School, and Collaborative Innovation Center for Biotherapy, Sichuan University, Chengdu 610041, China; 2 National Chengdu Center for Safety Evaluation of Drugs, State Key Laboratory of Biotherapy, West China Hospital, West China Medical School, and Collaborative Innovation Center for Biotherapy, Sichuan University, Chengdu 610041, China; 3 Analytical & Testing Center, Sichuan University, Chengdu 610041, China; 4 Department of Gastroenterology, West China Hospital, West China Medical School, Sichuan University, Chengdu 610041, China; 5 Department of Gastrointestinal Surgery, West China Hospital, West China Medical School, Sichuan University, Chengdu 610041, China

**Keywords:** cytoplasmic SHMT2, colorectal cancer, β-catenin, nonmetabolic function, ubiquitylation-mediated degradation

## Abstract

**Introduction:** Serine hydroxymethyltransferase 2 (SHMT2) plays a critical role in serine-glycine metabolism to drive cancer cell proliferation. However, the nonmetabolic function of SHMT2 in tumorigenesis, especially in human colorectal cancer (CRC) progression, remains largely unclear.

**Methods:** SHMT2 expression in human CRC cells was identified by western blot and immunofluorescence assay. The CRC cell proliferation, migration, and invasion after SHMT2 knockdown or overexpression were explored through *in vitro* and *in vivo* assays. Immunofluorescence, mRNA-seq, co-immunoprecipitation, chromatin immunoprecipitation-qPCR and immunohistochemistry assays were used to investigate the underlying mechanisms behind the SHMT2 nonmetabolic function.

**Results:** We demonstrated that SHMT2 was distributed in the cytoplasm and nucleus of human CRC cells. SHMT2 knockdown resulted in the significant inhibition of CRC cell proliferation, which was not restored by serine, glycine, or formate supplementation. The invasion and migration of CRC cells were suppressed after SHMT2 knockdown. Mechanistically, SHMT2 interacted with β-catenin in the cytoplasm. This interaction inhibited the ubiquitylation-mediated degradation of β-catenin and subsequently modulated the expression of its target genes, leading to the promotion of CRC cell proliferation and metastasis. Notably, the lysine 64 residue on SHMT2 (SHMT2^K64^) mediated its interaction with β-catenin. Moreover, transcription factor TCF4 interacted with β-catenin, which in turn increased *SHMT2* expression, forming an SHMT2/β-catenin positive feedback loop. *In vivo* xenograft experiments confirmed that SHMT2 promoted the growth and metastasis of CRC cells. Finally, the level of SHMT2 was found to be significantly increased in human CRC tissues. The SHMT2 level was correlated with an increased level of β-catenin, associated with CRC progression and predicted poor patient survival.

**Conclusion:** Taken together, our findings reveal a novel nonmetabolic function of SHMT2 in which it stabilizes β-catenin to prevent its ubiquitylation-mediated degradation and provide a potential therapeutic strategy for CRC therapy.

## Introduction

Metabolic regulation is essential for maintaining cancer cell proliferation, migration, and differentiation during cancer progression, and metabolic enzymes play a vital role in this process 1. Metabolic enzymes can be regulated at the gene and protein expression levels in response to extracellular and intracellular signaling to cope with the metabolic needs of cancer cells. Increasing evidence has demonstrated that metabolic enzymes can, directly or indirectly, modify the gene and protein expression of oncogenes and thereby influence cancer progression 2. For instance, pyruvate kinase M2 isoform (PKM2), a rate-limiting metabolic enzyme that catalyzes the final step of glycolysis, interacts with Bcl-2 and β-catenin, regulating their expression or activity to promote tumor growth 3, 4. These studies indicate that metabolic enzymes participate in tumor progression independent of their canonical metabolic roles. However, the nonmetabolic functions and underlying mechanisms by which they regulate cancer progression remain largely unknown.

Serine hydroxymethyltransferase 2 (SHMT2) is a key metabolic enzyme that converts serine to glycine. This reaction represents a major source of methyl groups for the one-carbon pools required for *de novo* nucleotide biosynthesis and DNA methylation 5. SHMT2 overexpression is observed in various cancers, including breast cancer, melanoma, lung cancer, ovarian cancer, and prostate cancer, and is associated with tumorigenesis and progression 6-8. In breast cancer, SHMT2 upregulation leads to an increased concentration of nicotinamide adenine dinucleotide phosphate (NADPH) and improves redox balance, which in turn facilitates cancer cell growth under hypoxic conditions 9. In non-small-cell lung cancer (NSCLC), the transcriptional upregulation of SHMT2 by NRF2 supports the production of glutathione and nucleotides, which correlates with poor prognosis in NSCLC patients 10. The above studies mainly explored the catalytic functions of SHMT2, which is located in mitochondria and works as a metabolic enzyme. However, several studies have indicated that SHMT2 is also found in the cytoplasm and nucleus and exhibits other biological functions apart from metabolic enzymatic activity, an idea that has recently begun to be appreciated. Anderson et al. found that the *SHMT2* gene encodes two transcripts: the first transcript encodes a well-known protein, mitochondrial SHMT2, while the second transcript lacks the mitochondrial import sequence and encodes a protein that localizes to the cytoplasm and nucleus. *SHMT2* encodes proteins that localize to the cytoplasm, nucleus, and mitochondria, suggesting potential new roles of this protein in the cytoplasm 11. There have been few studies about the nonenzymatic functions of SHMT2. Cytoplasmic SHMT2 directs BRCC36 isopeptidase complex (BRISC) activity at K63-Ub chains combined with the type 1 interferon receptor chain 1 (IFNAR1). BRISC-SHMT2 complexes localize to and deubiquitinate IFNAR1, which limited IFNAR1 internalization and regulated immune signals 12-14. Cao et al. found that cytoplasmic SHMT2 was identified as a defatty-acylation substrate of histone deacetylase 11 (HDAC11). HDAC11-catalyzed defatty-acylation did not affect the enzymatic activity of SHMT2 but affected its ability to regulate IFNAR1 ubiquitination, thus modulating immune responses 15. The above studies motivated our interest to further elucidate the multiple functions of SHMT2 other than its enzymatic activity, though the above studies are not associated with cancer progression. However, the function and mechanism underlying the nonmetabolic activity of SHMT2 in cancer remains largely unclear.

Colorectal cancer (CRC) is the third most commonly diagnosed malignancy and the second leading cause of cancer-related death worldwide 16. Although therapeutic strategies such as surgery, neoadjuvant chemotherapy, and targeted drugs have been employed over the past decades, the overall survival rate of patients with CRC is still far from satisfactory, especially for advanced CRC cases 17. Most patients identified with CRC are already in the advanced stage, and approximately 25% of patients with CRC present with liver metastases at the time of initial diagnosis 18. Most CRC patients eventually die due to tumor metastasis or recurrence after routine treatment. Therefore, a better understanding of the mechanism involved in CRC progression and metastasis is urgently needed. Emerging studies have suggested the involvement of SHMT2 in CRC progression. Mitochondrial SHMT2 K95 acetylation is frequently downregulated in human CRC tissues and is inversely associated with increased SIRT3 expression, which is correlated with poor overall postoperative survival in CRC patients 19. Though these studies suggest the involvement of SHMT2 in CRC development, the role of SHMT2 in CRC progression and metastasis and the underlying mechanism is not fully understood.

The purpose of the present study was to explore the role of SHMT2 in the progression and metastasis of CRC, particularly focusing on the nonmetabolic function of SHMT2 in CRC. Our results showed that cytoplasmic *SHMT2* acts as an oncogene promoting CRC cell progression and metastasis by inhibiting the degradation of β-catenin. SHMT2^K64^ is the key amino acid mediating its interaction with β-catenin. Furthermore, *SHMT2* is a target gene of TCF4/β-catenin. Taken together, our studies provide evidence that cytoplasmic SHMT2 plays an important role in promoting CRC progression by inhibiting the degradation of β-catenin, implicating SHMT2 as both a potential therapeutic target and a predictor of survival in CRC.

## Results

### SHMT2 is located in the cytoplasm and nucleus in addition to the mitochondria in human CRC cells

To explore the expression of SHMT2 in CRC, we profiled the mRNA and protein expression of SHMT2 in a panel of human CRC cells and the human normal colonic epithelial cells NCM460 and HCoEpiC. The expression of SHMT2 was generally high in HCT116 and DLD-1 cells and low in HT-29 cells (Figure S1A-B). Thus, HCT116, DLD-1, and HT-29 cells were used in subsequent studies.

**Figure 1:**
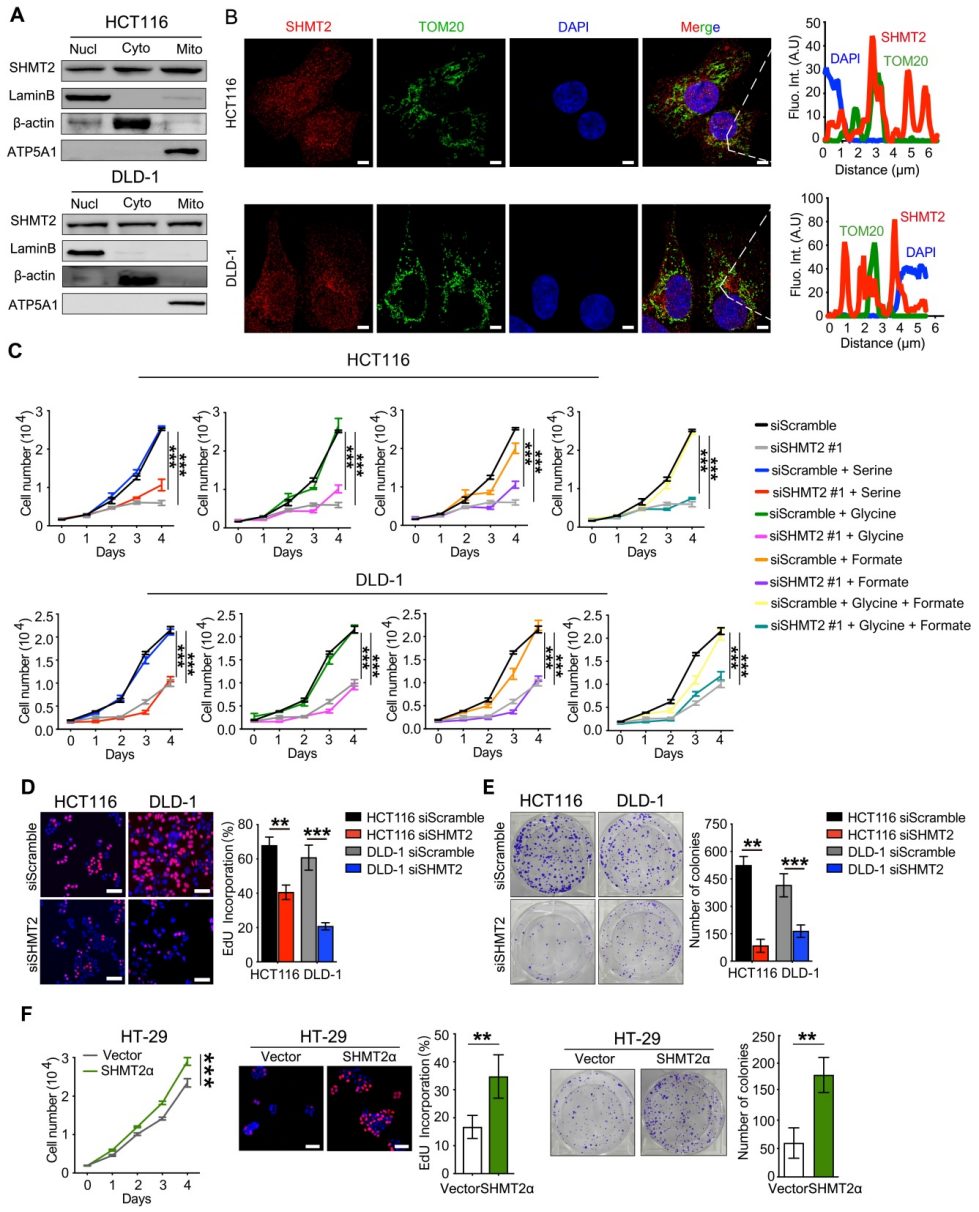
** SHMT2 is located in the cytoplasm and nucleus in addition to the mitochondria in human CRC cells and promotes cells proliferation.** (A) Cytoplasmic, mitochondrial, and nuclear fractions of human CRC cells were prepared, and western blot analysis was performed to detect SHMT2 expression. (Lamin B1: nuclear marker; β-actin: cytoplasmic marker; ATP5A1: mitochondrial marker). (B) Representative immunofluorescence images (left) of SHMT2 in HCT116 and DLD-1 cells. Merged images represent overlays of TOM20 (green), SHMT2 (red), and the nucleus (stained by DAPI, blue). The fluorescence intensities (right) of each pixel along the white lines crossing the cells were calculated to show the incomplete colocalization of SHMT2 with TOM20. Scale bar, 5 μm. (C) Cell proliferation was detected by CCK8 assay after SHMT2 knockdown with or without 400 μM serine, 400 μM glycine, or 1mM formate supplementation (n = 3). *** *p* < 0.001; ordinary one-way ANOVA multiple comparisons test. (D) - (E) Representative images (left) and quantification (right) of EdU incorporation assay (D) (n = 5) and colony formation assay (E) (n = 3) using HCT116 and DLD-1 cells with SHMT2 knockdown. Scale bar, 50 μm. ** *p* < 0.01, *** *p* < 0.001; Student's t-test. (F) Representative images and quantification of cell proliferation (left) (n = 3), EdU incorporation assay (middle) (n = 5) and colony formation assay (right) (n = 3) in HT29 cells with SHMT2α overexpression. Scale bar, 50 μm. ** *p* < 0.01, *** *p* < 0.001; Student's t-test. The data are presented as the mean ± SD from at least three independent experiments. The error bars show the SD.

We then determined the subcellular localization of SHMT2 in CRC cells. Western blot analysis showed that SHMT2 was predominantly localized in the cytoplasm and nucleus in addition to the mitochondria in CRC cells (Figure 1A). Similar distribution patterns were observed in the immunofluorescence (IF) assay (Figure 1B). The expression curve analysis showed that SHMT2 expression (red) coincided with the expression of TOM20 (green), a mitochondrial marker, and with the expression of DAPI (blue), a nuclear marker (Figure 1B). Collectively, these data indicated that SHMT2 is located in the cytoplasm and nucleus of human CRC cells in addition to the mitochondria.

### SHMT2 regulates human CRC cell proliferation and apoptosis *in vitro*

To investigate the potential functional roles of SHMT2 in CRC, we employed siRNAs to knockdown SHMT2 expression in HCT116 and DLD-1 cells. Successful SHMT2 knockdown in cells was confirmed by RT-qPCR and western blot analysis (Figure S1C). In addition, SHMT2^WT^ and SHMT2α, an isoform of SHMT2 that lacks the mitochondrial localization residues (N-terminal 1-21 amino acids), were successfully overexpressed in HT-29 cells through lentiviral-mediated transfection, respectively (Figure S1D).

The effect of SHMT2 on cell proliferation was investigated by the cell counting kit-8 (CCK-8) assay. SHMT2 knockdown led to significant proliferation inhibition in HCT116 and DLD-1 cells (Figures 1C and S2A). The serine and glycine levels in CRC cells after SHMT2 knockdown were measured by liquid chromatography-mass spectrometry (LC-MS). The results showed that the serine level was significantly increased and the glycine level was decreased by 50% compared with control cells (Figure S2B), indicating that SHMT2 knockdown inhibited glycine synthesis. Notably, additional supplementation with serine, glycine, and formate did not significantly restore the proliferation inhibition induced by SHMT2 knockdown, suggesting that there might be a nonenzymatic function of SHMT2 that is involved in the proliferation of cells in addition to its enzymatic function (Figures 1C and S2A).

The 5-ethynyl-2'-deoxyuridine (EdU) incorporation assay and the colony formation assay further confirmed that SHMT2 knockdown suppressed CRC cell proliferation (Figure 1D-E). Conversely, the exogenous expression of SHMT2^WT^ led to the promotion of proliferation in HT-29 cells (Figure S2C), and similar results were obtained when SHMT2α was expressed in HT-29 cells (Figure 1F). These results suggest that SHMT2 overexpression is necessary to support CRC cell proliferation. Moreover, SHMT2 knockdown in HCT116 and DLD-1 cells significantly increased the number of annexin V/PI-positive cells compared with that in the control group (Figure S2D). Furthermore, the protein levels of cleaved caspase-3, Bax, and Bcl-2, which are markers of apoptosis, were evaluated by western blot. The results showed that upregulation of the proapoptosis proteins Bax and cleaved caspase-3 and downregulation of the antiapoptosis protein Bcl-2 were observed in CRC cells after SHMT2 knockdown (Figure S2E). Conversely, SHMT2^WT^ and SHMT2α overexpression in HT-29 cells resulted in the downregulation of Bax and cleaved caspase-3 and upregulation of Bcl-2 (Figure S2E). Taken together, these observations indicate that SHMT2 promotes CRC cell proliferation and that the deletion of SHMT2 severely disrupts this proliferation.

### SHMT2 promotes the migration and invasion of CRC cells *in vitro*

**Figure 2:**
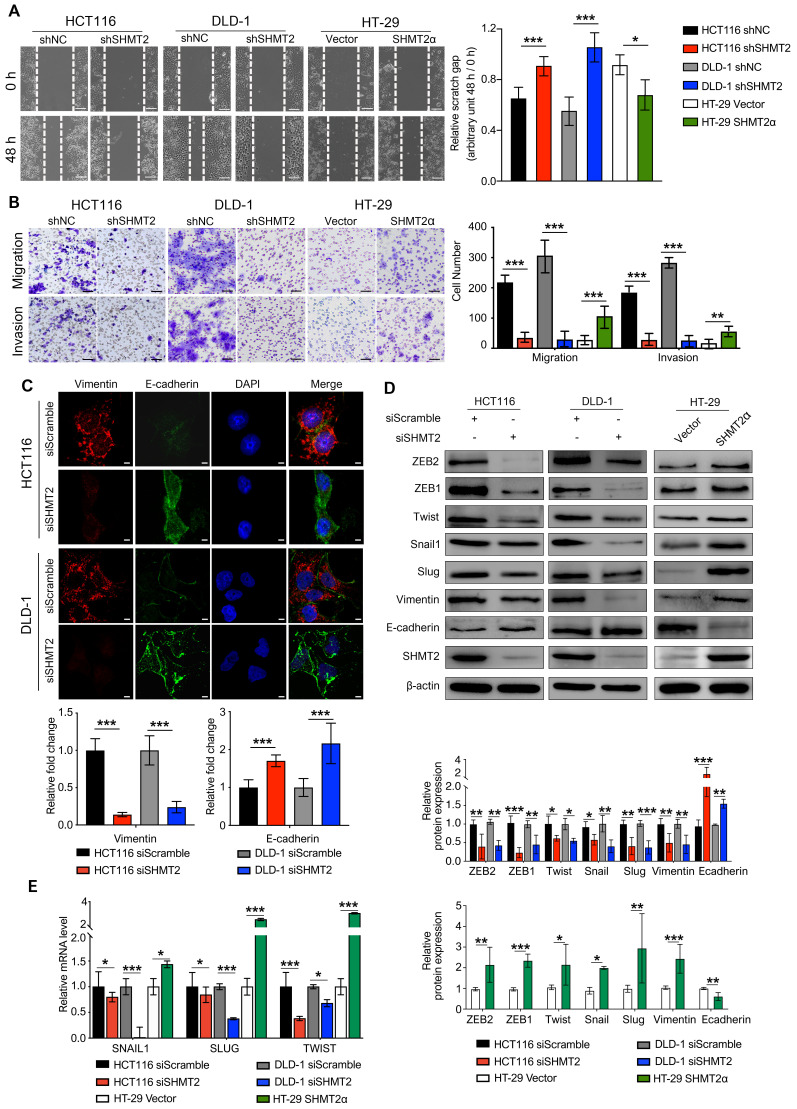
** SHMT2 promotes the migration and invasion of CRC cells *in vitro.***(A) Representative images (left) and quantification (right) of the wound healing assay using CRC cells with SHMT2 knockdown or SHMT2α overexpression (n = 3). Scale bar, 200 μm. * *p* < 0.05, *** *p* < 0.001; Student's t-test. (B) Representative images (left) and quantification (right) of the migration and invasion assay using CRC cells with SHMT2 knockdown or SHMT2α overexpression (n = 3). Scale bar, 100 μm. ** *p* < 0.01, *** *p* < 0.001; Student's t-test. (C) Representative immunofluorescence images (up) and quantification (down) of vimentin and E-cadherin in CRC cells. Merged images represent overlays of vimentin (red), E-cadherin (green), and nucleus (blue). Scale bar, 5 μm. *** *p* < 0.001; Student's t-test. (D) Western blot analysis (up) and quantification (down) of vimentin, E-cadherin, and EMT-associated transcription factors in CRC cells after SHMT2 knockdown or SHMT2α overexpression. * *p* < 0.05, ** *p* < 0.01, *** *p* < 0.001; Student's t-test. (E) RT-qPCR analysis of EMT-associated transcription factors in CRC cells after SHMT2 knockdown or SHMT2α overexpression (n = 3). * *p* < 0.05, *** *p* < 0.001; Student's t-test. The data are presented as the mean ± SD from at least three independent experiments. The error bars show the SD.

Metastasis is the leading cause of CRC mortality, and the migration and invasion of cancer cells are critical steps in cancer metastasis. To explore whether SHMT2 drives migration and invasion, we conducted wound healing, Transwell, and Matrigel invasion assays using CRC cells with SHMT2 knockdown or overexpression (Figure S3A). The results showed that SHMT2 knockdown significantly weakened the migration and invasion of HCT116 and DLD-1 cells (Figure 2A-B). In addition, HT-29 cells with low SHMT2 expression showed weak migration and invasion ability, while the overexpression of SHMT2^WT^ and SHMT2α enhanced the migration and invasion ability of HT-29 cells (Figures 2A-B, S3B-C). As SHMT2α is located in the cytoplasm, these data suggested that a nonenzymatic function of SHMT2 is involved in the migration and invasion properties.

Lysine 95 and lysine 280 are the key amino acids for SHMT2 enzymatic activity 19, 20. To further confirm this speculation, we overexpressed SHMT2^K95A^ and SHMT2^K280A^ in HT-29 cells (Figure S3D). And the cell migration and invasion were evaluated. SHMT2^K95A^ and SHMT2^K280A^ overexpression also significantly increased the migration and invasion ability of HT-29 cells (Figure S3E-F). These data suggest that SHMT2 promotes CRC cell migration and invasion *in vitro*, and the nonenzymatic function of SHMT2 is important for the migration and invasion ability of CRC cells.

Given the essential role of epithelial-mesenchymal transition (EMT) in tumor metastasis 21, we used IF to investigate the expression of vimentin and E-cadherin, two important EMT markers. The results showed that SHMT2 knockdown significantly downregulated vimentin expression and upregulated E-cadherin expression in HCT116 and DLD-1 cells (Figure 2C). Similar results were obtained in the western blot analysis (Figure 2D). In contrast, the overexpression of SHMT2^WT^ and SHMT2α in HT-29 cells downregulated E-cadherin and upregulated vimentin (Figures 2D and S3G). We further evaluated the expression of a panel of EMT-associated transcription factors, including Snail, Slug, TWIST, ZEB1, and ZEB2. SHMT2 knockdown significantly decreased the protein levels of these EMT-associated transcription factors in HCT116 and DLD-1 cells, whereas SHMT2^WT^ and SHMT2α overexpression in HT-29 cells led to the opposite results (Figures 2D and S3G). In addition, the mRNA levels of EMT-associated transcription factors decreased after SHMT2 knockdown and increased after SHMT2^WT^ and SHMT2α overexpression (Figures 2E and S3H). Collectively, our results show that SHMT2 induces EMT in CRC cells by regulating EMT-associated transcription factors.

### SHMT2 depletion impairs the Wnt/β-catenin pathway

**Figure 3:**
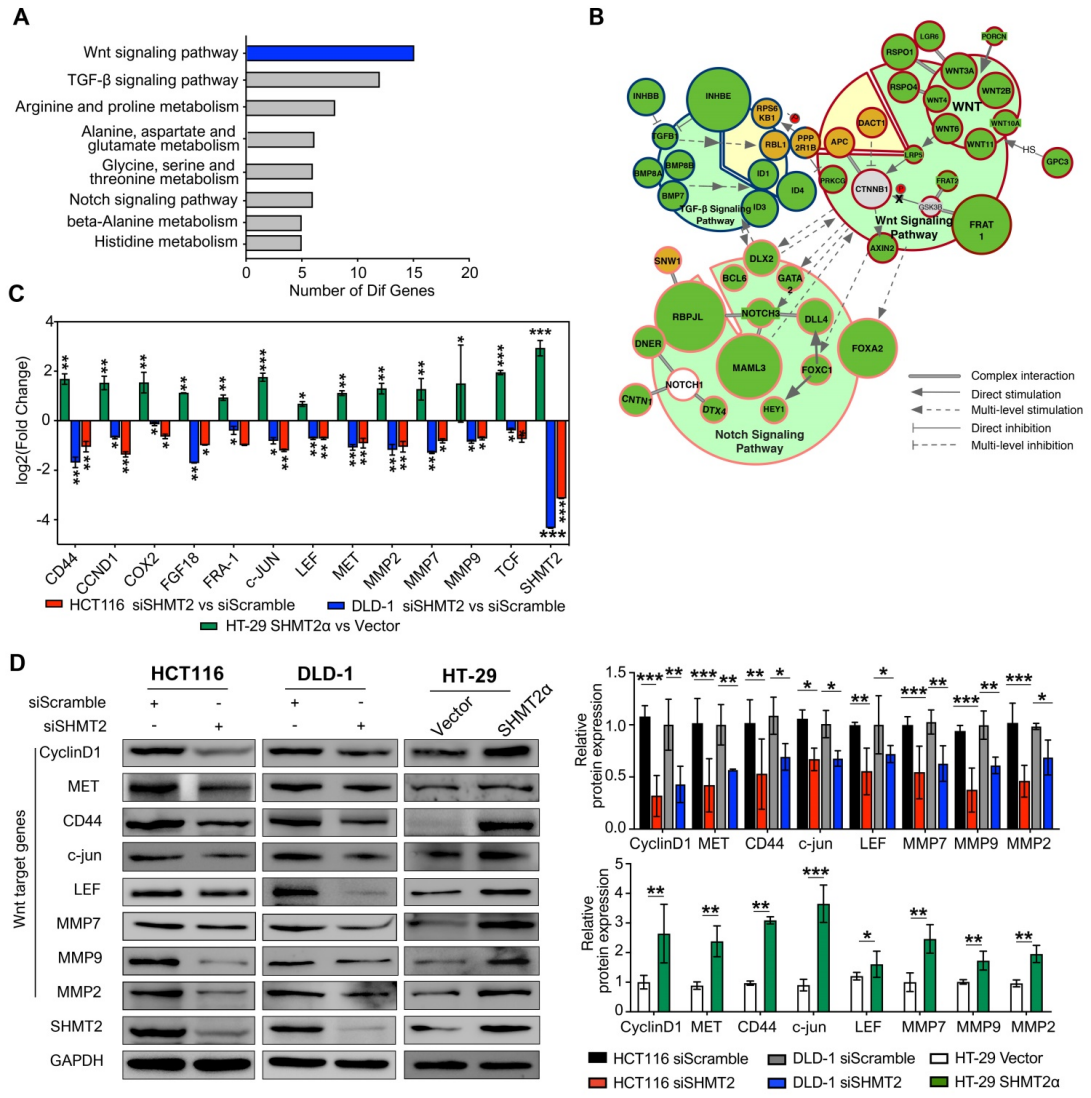
** SHMT2 depletion impairs the Wnt/β-catenin pathway.** (A) Representative GO term analysis of downregulated genes after SHMT2 knockdown. (B) The gene interactions of the TGF-β, Notch, and Wnt signaling pathways. Different stroke colors represent different signaling pathways. Green, yellow, and gray represent downregulation, upregulation, and no significant change, respectively. The radius of the dots indicates the amplitude variation. (C) RT-qPCR analysis of Wnt target genes with SHMT2 knockdown or SHMT2α overexpression (n = 3). * *p* < 0.05, ** *p* < 0.01, *** *p* < 0.001; Student's t-test. (D) Western blot analysis (left) and quantification (right) of Wnt target genes in CRC cells with SHMT2 knockdown or SHMT2α overexpression. * *p* < 0.05, ** *p* < 0.01, *** *p* < 0.001; Student's t-test. The data are presented as the mean ± SD from at least three independent experiments. The error bars show the SD.

To gain insight into the mechanism by which SHMT2 regulates proliferation and migration in CRC cells, we applied mRNA sequencing technology to explore the factors regulated by SHMT2. The results showed that SHMT2 knockdown profoundly altered the expression of almost 200 genes in HCT116 cells (Figure S4A-B). According to the Gene Ontology (GO) Consortium, SHMT2 knockdown clearly suppressed multiple signaling pathways, including the TGF-β, Notch, and Wnt pathways, which are closely related to CRC progression (Figure 3A). Among them, the number of genes that changed in the Wnt signaling pathway was the largest (Figure 3A, Table S1). Furthermore, cell signaling network analysis of these significantly altered genes revealed that Wnt/β-catenin signaling was critical in these pathways and closely connected with the TGF-β and Notch pathways (Figure 3B). Importantly, mRNA-seq assays showed that SHMT2 knockdown decreased the mRNA levels of multiple target genes of the Wnt/β-catenin pathway, such as* c-Myc*, *CD44*, *ID2*, *L1CAM*, and *RUNX2.* We then performed RT-qPCR assays to verify the representative genes obtained from the mRNA-seq results. The expression of the oncogenes *c-Myc* and* CCND1*, which are closely involved in the Wnt/β-catenin pathway, was decreased along with SHMT2 knockdown. Simultaneously, the tumor-suppressor genes *p21*,* PTEN*, and *TGFBR2* were upregulated (Figure S4C).

To further investigate the effect of SHMT2 on Wnt/β-catenin signaling, we measured the mRNA levels of β-catenin target genes important in CRC progression. SHMT2 knockdown significantly decreased the levels of these β-catenin targets, including *CD44*, *CCND1*, *COX-2*, *FGF18*, *MMP2*, *MMP7* and *MMP9* (Figure 3C). Western blot analysis further confirmed that SHMT2 knockdown markedly suppressed the expression of β-catenin targets in HCT116 and DLD-1 cells (Figure 3D). Conversely, SHMT2α and SHMT2^WT^ overexpression enhanced the mRNA and protein levels of these Wnt/β-catenin targets (Figures 3C-D and S4D-E).

### SHMT2 maintains β-catenin stability by inhibiting its ubiquitylation

**Figure 4:**
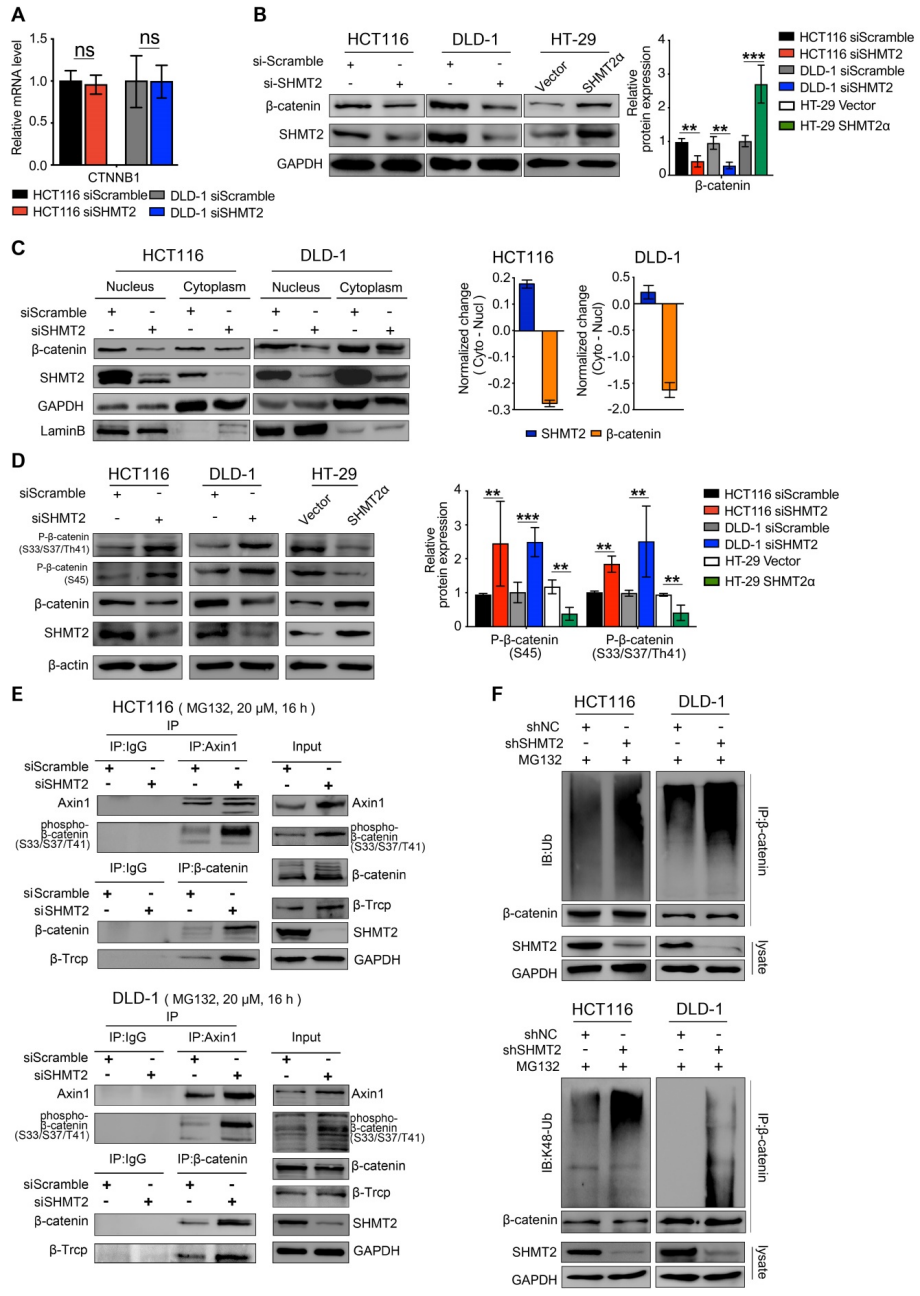
** SHMT2 maintains β-catenin stability by inhibiting its degradation.** (A) RT-qPCR analysis of β-catenin in HCT116 and DLD-1 cells with siSHMT2 (n = 3). Ns: not significant; Student's t-test. (B) Western blot analysis (left) and quantification (right) of β-catenin in CRC cells after SHMT2 knockdown or SHMT2α overexpression. ** *p* < 0.01, *** *p* < 0.001; Student's t-test. (C) Western blot analysis (left) of SHMT2 and β-catenin after cell fractionation in HCT116 and DLD-1 cells with siSHMT2. Comparison of the relative reduction (right) of SHMT2 and β-catenin proteins in the cytoplasm and nucleus in CRC cells. (D) Western blot analysis (left) and quantification (right) of phosphorylated β-catenin after SHMT2 knockdown or SHMT2α overexpression in CRC cells. ** *p* < 0.05, *** *p* < 0.001; Student's t-test. (E) HCT116 (up) and DLD-1 cells (down) were treated with MG132 for 16 h and with siSHMT2 for 48 h. Cell proteins were subjected to immunoprecipitation using the indicated antibodies. (F) CRC cells stably expressing shNC or shSHMT2 were treated with MG132 for 16 h before harvesting. β-Catenin was immunoprecipitated and immunoblotted with the indicated antibodies. The data are presented as the mean ± SD from at least three independent experiments. The error bars show the SD.

Because SHMT2 knockdown downregulated Wnt/β-catenin target genes, we investigated β-catenin, a major transducer of the Wnt-mediated cascade. To this end, we detected the expression of β-catenin in CRC cells after SHMT2 knockdown. SHMT2 knockdown did not alter β-catenin mRNA levels (Figure 4A). Interestingly, the protein level of β-catenin was significantly decreased after SHMT2 knockdown and increased after SHMT2^WT^ and SHMT2α overexpression (Figures 4B and S5A). We further investigated the protein level of β-catenin in the nucleus and cytoplasm. SHMT2 knockdown markedly suppressed the β-catenin protein level in both the nucleus and cytoplasm (Figure 4C). The grayscale analysis method was used to quantify the changes of SHMT2 and β-catenin in the cytoplasm and nucleus caused by SHMT2 knockdown. The decrease in SHMT2 was more obvious in the cytoplasm, while the reduction of β-catenin mainly occurred in the nucleus (Figure 4C). These data suggest that SHMT2 knockdown results in decreased β-catenin protein levels, especially nuclear β-catenin.

Since SHMT2 knockdown decreased the protein level of β-catenin without changing the mRNA level, we speculated that SHMT2 knockdown might regulate the β-catenin protein level by posttranslational modification. Ubiquitylation degradation is a key step of β-catenin posttranslational modification. The phosphorylation of β-catenin serine 33 and 37 creates a binding site for the E3 ubiquitin ligase β-TrCP, leading to β-catenin ubiquitination and degradation 22. Considering that elevated levels of phosphorylated β-catenin are a prerequisite for its ubiquitination, we detected the phosphorylation level of β-catenin. SHMT2 knockdown markedly increased the phosphorylation of β-catenin at both the Ser45 residue and the Ser33/37Thr41 residues in HCT116 and DLD-1 cells (Figure 4D). In contrast, SHMT2^WT^ and SHMT2α overexpression in HT-29 cells significantly reduced the level of phospho-β-catenin (Figures 4D and S5B).

Considering that the assembly of Axin1 is the rate-limiting step of β-catenin ubiquitination, we examined the interaction of Axin1 with phospho-β-catenin after SHMT2 knockdown. The results showed that phosphorylated β-catenin was apparently bound to Axin1 in SHMT2-knockdown cells (Figure 4E lanes 3, 4). We continued to investigate whether SHMT2 could modulate β-catenin degradation through β-TrCP-mediated ubiquitination. As expected, the co-immunoprecipitation (Co-IP) assay showed binding between β-TrCP and β-catenin in both HCT116 and DLD-1 cells upon SHMT2 knockdown (Figure 4E lanes 3, 4).

Finally, we examined the β-catenin ubiquitination level in CRC cells after SHMT2 deletion. We found significant co-immunoprecipitation of β-catenin with K48 ubiquitin after SHMT2 knockdown, suggesting that SHMT2 knockdown enhanced the ubiquitination of β-catenin in CRC cells (Figure 4F).

Given that SHMT2 and BRCC36 complexes work together to promote K-63 ubiquitin deubiquitination 13, we investigated whether BRCC36 is involved in β-catenin deubiquitination. By knocking down BRCC36 expression using a specific siRNA in both HCT116 and DLD-1 cells, we found that the protein expression of β-catenin was reduced after BRCC36 knockdown (Figure S5C). Moreover, SHMT2 knockdown resulted in significant co-immunoprecipitation of β-catenin with K63 ubiquitin, suggesting that SHMT2 enhanced the BRCC36-mediated deubiquitination of β-catenin in CRC cells (Figure S5D). Collectively, these results indicate that SHMT2 knockdown enhances β-catenin ubiquitylation, which triggers the degradation of β-catenin.

### The K64 residue of SHMT2 is required for its interaction with β-catenin in CRC cells

**Figure 5:**
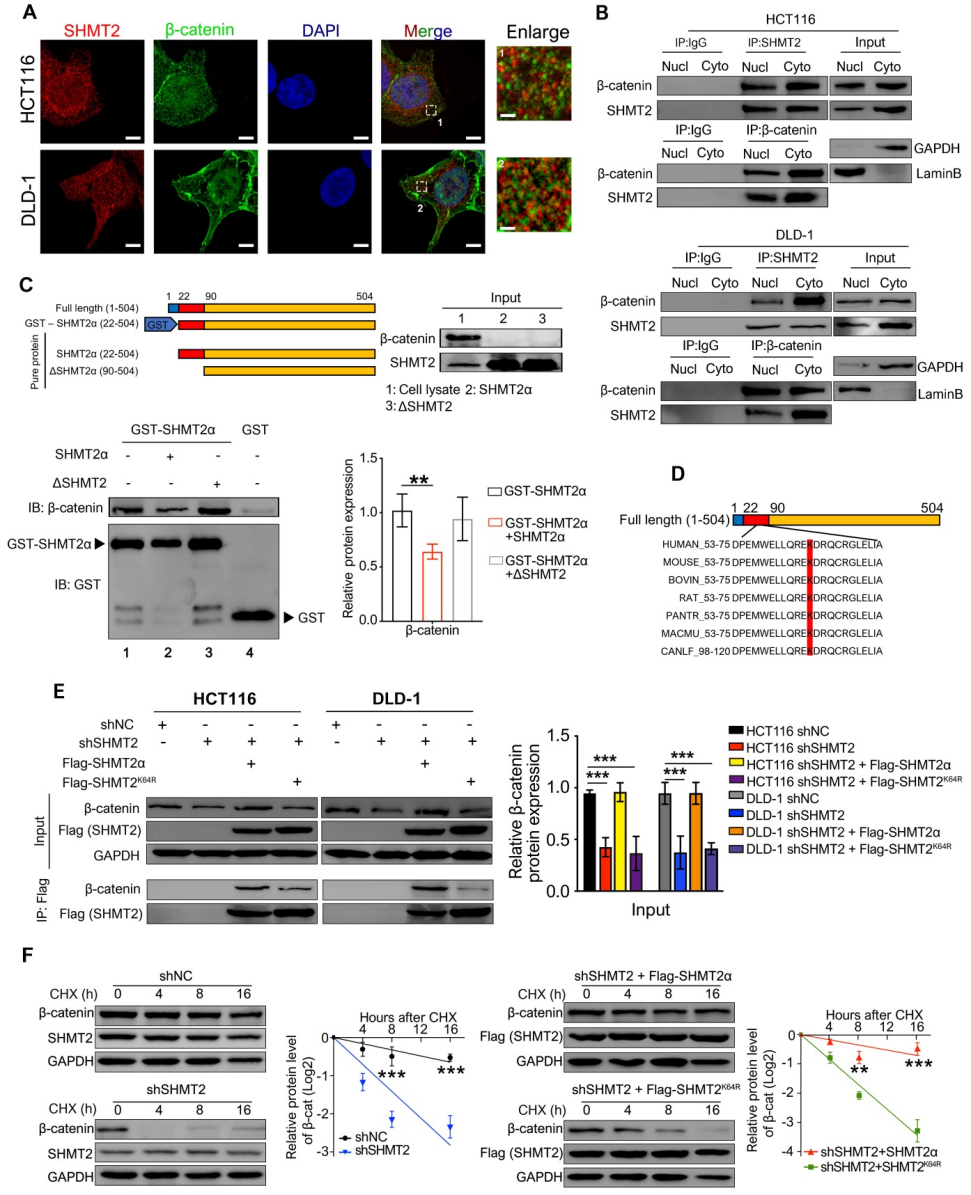
** SHMT2 interacts with β-catenin at K64.** (A) Representative immunofluorescence images of SHMT2 (blue) and β-catenin (red) in HCT116 and DLD-1 cells. Scale bars, 5 μm, 1 μm. (B) The cytoplasmic and nuclear protein was separated from HCT116 and DLD-1 cells and subjected to immunoprecipitation using the indicated antibodies. (C) Pure SHMT2 protein with the different indicated constructions were added into the cell lysates derived from HCT116 cells. Immunoblot analysis of GST pull-down using the indicated antibodies. The relative β-catenin expression (normalized to GST-SHMT2α) was quantified. ** *p* < 0.01; Student's t-test. (D) The alignment of SHMT2 for human, mouse, bovin, rat, pantr, macmu, and canlf, shows highly conserved residues in SHMT2, including lysine 64. (E) Stable SHMT2 knockdown HCT116 and DLD-1 cells that re-expressed the shRNA-resistant SHMT2α or K64R mutation were constructed. The cells were subjected to immunoprecipitation using the indicated antibodies. (F) Western blot analysis (left) and quantification (right) of β-catenin in HCT116 cells with stable SHMT2 knockdown that re-expressed shRNA-resistant SHMT2α or K64R mutation after cycloheximide (CHX) treatment at different time points. ** *p* < 0.01, *** *p* < 0.001; Student's t-test. The data are presented as the mean ± SD from at least three independent experiments. The error bars show the SD.

Because our results suggested that SHMT2 knockdown triggers robust phosphorylation-dependent ubiquitination of β-catenin, we inferred that the colocalization of SHMT2 and β-catenin should be a precondition of these observations. Therefore, IF was used to analyze whether SHMT2 was colocalized with β-catenin in CRC cells. SHMT2 was found to partially colocalize with β-catenin in both HCT116 and DLD-1 cells (Figure 5A). We then performed Co-IP experiments to confirm the interaction between endogenous SHMT2 and β-catenin. Strong coprecipitation of endogenous SHMT2 and β-catenin was detected in the cytoplasm, suggesting that SHMT2 can interact with β-catenin in HCT116 and DLD-1 cells (Figure 5B). These results support our observation that SHMT2 regulates β-catenin degradation by interacting with β-catenin.

To further understand the molecular basis by which SHMT2 interacts with β-catenin, we searched for specific sites in SHMT2 isoforms that respond to β-catenin. Sequence analysis was performed based on the UniProt database (https://www.uniprot.org/uniprot/P34897), and the results indicated the presence of three main SHMT2 protein isoforms in mammalian cells, which have more than 95% identical residues. Isoform 3 (SHMT2α) showed a deficiency of N-terminal aa 1-21, suggesting that isoform 3 lacks the mitochondrial localization residues and is involved in the deubiquitination process. We generated GST-tagged SHMT2α (GST-SHMT2α), SHMT2α and SHMT2 deletion mutants lacking the first 3-layer alpha/beta/alpha sandwich topology at the N-terminus of SHMT2 (∆SHMT2) *in vitro* and performed the GST pull-down assay (Figure 5C). Our results showed that GST-SHMT2α strongly pulled down β-catenin in HCT116 cell lysates (Figure 5C, lane 1). Excess addition of SHMT2α protein into cell lysates caused less β-catenin binding with GST-SHMT2α (Figure 5C, lane 2). Notably, the ∆SHMT2 protein was unable to disturb GST-SHMT2α binding with β-catenin (Figure 5C, lane 3), indicating that the N-terminal aa 22-89 of SHMT2 is essential for its binding to β-catenin.

We next searched for specific sites in SHMT2 that are involved in the β-catenin interaction. Previous studies showed that lysine plays an important role in the function of SHMT2 19, 20, suggesting the necessity of lysine for SHMT2 structural function for deubiquitinating regulation. Thus, we investigated whether the lysine in the N-terminal 22-89 aa region would mediate the interaction between SHMT2 and β-catenin. The sequence analysis found only one lysine in the N-terminal 22-89 aa region, at residue 64 (SHMT2^K64^) (Figure 5D); Therefore, we created a Flag-tagged mutant with a lysine-to-arginine change at the 64^th^ residue (Flag-SHMT2^K64R^). The Flag-tagged SHMT2α and SHMT2^K64R^ were re-expressed in CRC-shSHMT2 cells. Notably, the re-expression of SHMT2α restored the expression of β-catenin in CRC-shSHMT2 cells. In contrast, the re-expression of SHMT2^K64R^ did not restore β-catenin expression, suggesting that K64 in SHMT2 plays a critical role in the interaction with β-catenin (Figure 5E). As expected, SHMT2α efficiently coprecipitated with β-catenin, while SHMT2^K64R^ lost the ability to interact with β-catenin (Figure 5E). To further test whether K64 of SHMT2 could regulate β-catenin degradation, we determined the protein half-life of β-catenin upon manipulation of SHMT2 in the presence of cycloheximide (CHX), a protein synthesis inhibitor. In HCT116-shSHMT2 cells, SHMT2α re-expression markedly extended the protein half-life of β-catenin compared with the re-expression of SHMT2^K64R^ (Figure 5F). Collectively, we conclude that SHMT2^K64^ is the key amino acid that interacts with β-catenin.

### The K64 residue of SHMT2 is critical for CRC cell proliferation, migration and invasion and depends on β-catenin

To further demonstrate that K64 is critical for SHMT2 biofunction, we detected the proliferation, migration, and invasion of CRC cells re-expressing Flag-tagged SHMTα or SHMT2^K64R^ after SHMT2 knockdown. The re-expression of SHMT2α restored the proliferation inhibition of CRC cells induced by SHMT2 knockdown, whereas the re-expression of SHMT2^K64R^ did not (Figure S6A-B). As expected, SHMT2α efficiently restored the migration and invasion of CRC-shSHMT2 cells. In contrast, the re-expression of SHMT2^K64R^ eliminated this ability, suggesting that K64 in SHMT2 plays a critical role in CRC cell proliferation and migration (Figure S6C-D).

**Figure 6:**
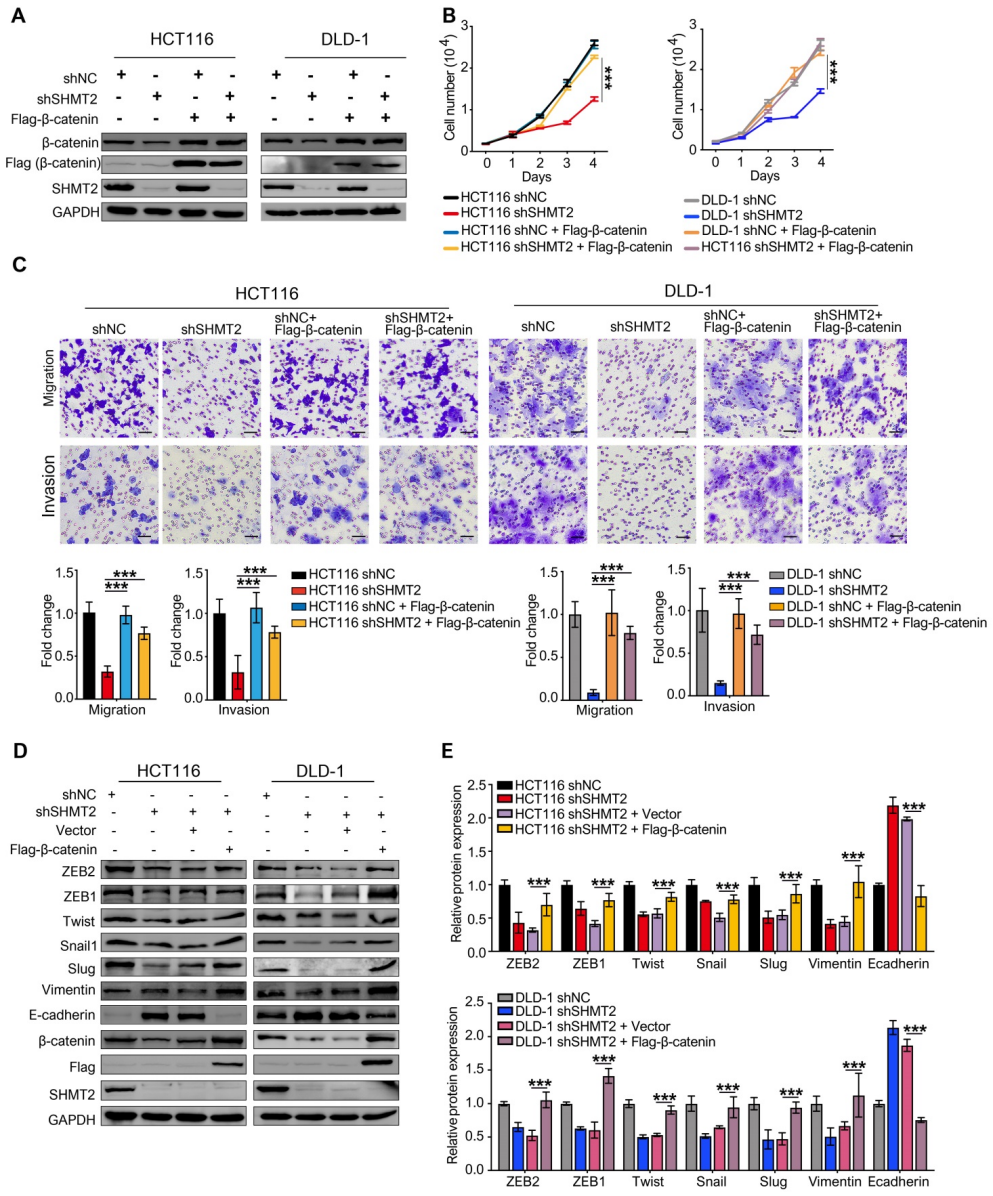
** SHMT2 is critical for CRC cell proliferation, migration, and invasion dependent on β-catenin.** (A) The overexpression of Flag-β-catenin in HCT116 and DLD-1cells after SHMT2 knockdown was confirmed by western blot analysis. (B) Cell proliferation was detected by CCK8 assay after stable SHMT2 knockdown with the re-expression of Flag-β-catenin (n = 3). *** *p* < 0.001; ordinary one-way ANOVA multiple comparisons test. (C) Representative images (top) of the Transwell migration and invasion assay using CRC cells after stable SHMT2 knockdown with the re-expression of Flag-β-catenin. The number of migrated cells was counted in three random fields per chamber and statistically analyzed (bottom). Scale bar, 100 μm. *** *p* < 0.001; ordinary one-way ANOVA multiple comparisons test. (D)-(E) Western blot analysis (D) and quantification (E) of vimentin, E-cadherin, and EMT-associated transcription factors in CRC cells with β-catenin overexpression after SHMT2 knockdown. * *p* < 0.05, ** *p* < 0.01, *** *p* < 0.001; ordinary one-way ANOVA multiple comparisons test. The data are presented as the mean ± SD from at least three independent experiments. Error bars are SD.

We also expressed β-catenin in CRC cells after SHMT2 knockdown and detected cell proliferation (Figure 6A). The overexpression of β-catenin significantly rescued the CRC cell proliferation inhibition induced by SHMT2 knockdown (Figure 6B). Similar results were observed in the migration and invasion assays (Figure 6C). Moreover, the overexpression of β-catenin significantly rescued the EMT gene downregulation induced by SHMT2 knockdown (Figure 6D). Taken together, these results suggest that SHMT2 regulates CRC cell proliferation, migration, and invasion by modulating β-catenin.

### SHMT2 is reversely regulated by β-catenin in CRC cells

Wnt signaling can promote the expression of several Wnt pathway components, indicating that feedback control is a key feature of Wnt signaling regulation. To investigate whether the Wnt/β-catenin pathway regulates SHMT2, SHMT2 was analyzed using PROMO software (http://alggen.lsi.upc.es). The data showed two putative binding sites of TCF4 located on the exon of the *SHMT2* gene, which were named site 1 (CTGTACT) and site 2 (AGCAAAG) (Figure S7A). We used a specific TCF4 antibody and RT-qPCR assay for chromatin immunoprecipitation (ChIP) in CRC cells. Remarkably, TCF4 binding at the open reading frame (ORF) of the *SHMT2* gene increased by 36-fold and 8-fold in CRC cells compared with control HCT116 and DLD-1 cells, respectively (Figure S7A). Because the transcription factor TCF4 acts as an effector of Wnt/β-catenin signaling, these findings suggest that Wnt/β-catenin may regulate SHMT2 expression. To test whether TCF4 regulates SHMT2 transcription by the Wnt/β-catenin pathway, CRC cells were treated with two inhibitors of Wnt pathway components, XAV939 (inhibiting Tankyrase, thereby stabilizing Axin1) and iCRT14 (disrupting the β-catenin-TCF4 interaction). iCRT14 treatment for 48 h significantly decreased the mRNA level of SHMT2 in a concentration-dependent manner in HCT116 and DLD-1 cells (Figure S7B). Western blot analysis further confirmed that XAV939 decreased SHMT2 expression and increased the phosphorylation of β-catenin at Ser33/37Thr41 in HCT116 and DLD-1 cells (Figure S7C). iCRT14 reduced the expression of SHMT2 and CyclinD1, a target gene of the Wnt/β-catenin pathway (Figure S7D). Consistent with these results, Wnt3a, a Wnt/β-catenin pathway activator, clearly upregulated SHMT2 expression in HT-29 cells (Figure S7E). Taken together, these results demonstrate that SHMT2 is a target gene of the Wnt/β-catenin pathway.

### SHMT2 promotes CRC cell growth and metastasis* in vivo*

**Figure 7:**
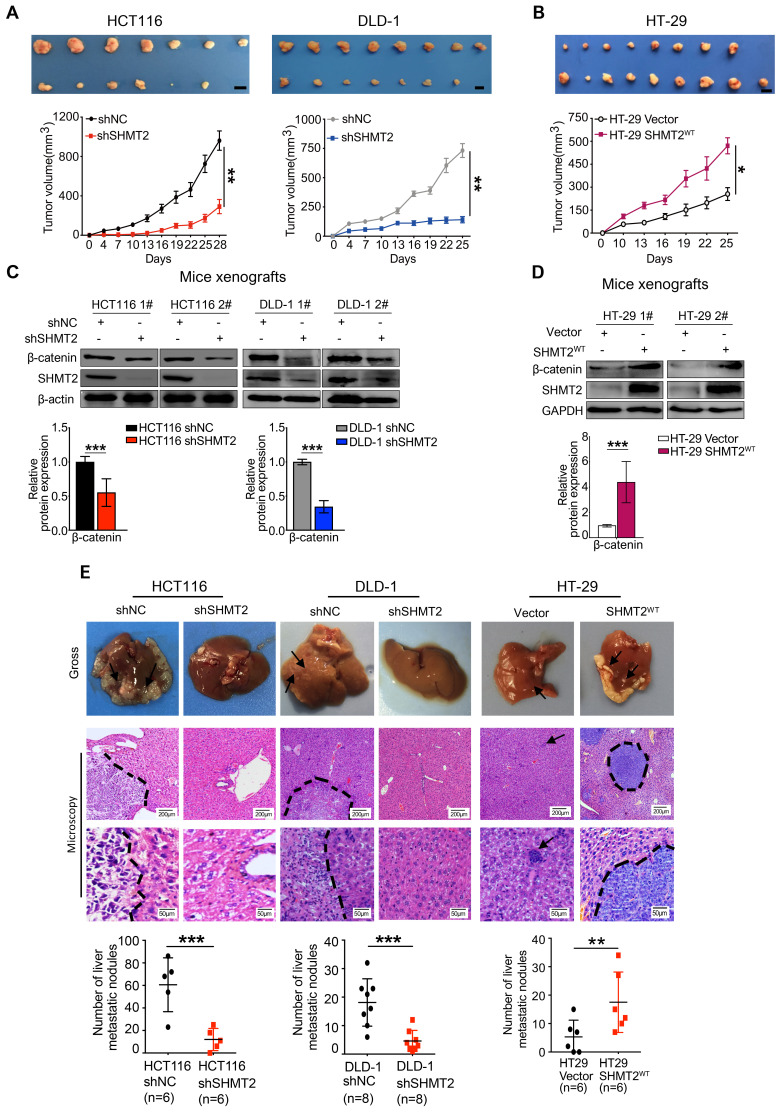
** SHMT2 promotes CRC cell growth and metastasis *in vivo.***(A) Representative images (up) and growth curves (down) of xenograft experiments using HCT116 (n = 7) and DLD-1 (n = 8) cells with SHMT2 knockdown (6-8 per group). Scale bar, 1 cm. * *p* < 0.05, ** *p* < 0.01; Student's t-test. The data are presented as the mean ± SEM from two independent experiments. The error bars show the SEM. (B) Representative images (up) and growth curve (down) of xenograft experiments using HT-29 cells with SHMT2^WT^ overexpression (n = 9). Scale bar, 1 cm. * *p* < 0.05, ** *p* < 0.01; Student's t-test. The data are presented as the mean ± SEM from two independent experiments. The error bars show the SEM. (C) Western blot analysis (top) and quantification (bottom) of SHMT2 and β-catenin in xenograft tumors from HCT116 and DLD-1 cells with SHMT2 knockdown. *** *p* < 0.001; Student's t-test. The data are presented as the mean ± SD from two independent experiments. The error bars show the SD. (D) Western blot analysis (top) and quantification (bottom) of SHMT2 and β-catenin in xenograft tumors from HT-29 cells with SHMT2^WT^ overexpression. *** *p* < 0.001; Student's t-test. The data are presented as the mean ± SD from two independent experiments. The error bars show the SD. (E) Representative gross (up) and microscopic (middle) images and the numbers of liver metastasis nodules (bottom) in an intrasplenic-nude mouse model of CRC cells with SHMT2 knockdown or overexpression. After 6 weeks, the mice were killed and the liver tissues were stained with H&E. The number of liver metastasis nodules was counted and statistically analyzed (n = 6-8 per group). Scale bar, 200 μm or 50 μm. ** *p* < 0.01, *** *p* < 0.001; Student's t-test. The data are presented as the mean ± SD from two independent experiments. The error bars show the SD.

To confirm the biological function of SHMT2 on tumor growth *in vivo*, xenograft experiments were performed by subcutaneously injecting shNC, HCT116-shSHMT2 cells and DLD-1-shSHMT2 cells into nude mice. SHMT2 knockdown significantly prevented tumor growth with an inhibition rate of 70-81% in both HCT116 and DLD-1 cell mouse models (Figure 7A). In contrast, SHMT2^WT^ overexpression in HT-29 cells obviously increased the tumor volume compared with that in controls (Figure 7B). Furthermore, western blot analysis revealed decreased levels of both β-catenin in tumor tissues derived from mice injected with HCT116 and DLD-1 cells with SHMT2 knockdown (Figure 7C). Conversely, an increased level of β-catenin was detected in tumor tissues derived from mice injected with HT-29 cells with SHMT2^WT^ expression, suggesting that the β-catenin expression level correlates with the SHMT2 level in mouse tumor tissues (Figure 7D).

In addition to the effect on tumor growth, the impact of SHMT2 on CRC metastasis was also evaluated *in vivo* using the intrasplenic-nude mouse model system (ISMS). Three paired cells, HCT116-shSHMT2/HCT116-shNC, DLD-1-shSHMT2/DLD-1-shNC, and HT-29 SHMT2^WT^/HT-29 vector, were injected into the spleen capsule of nude mice. Six weeks later, the mice were sacrificed, and liver metastatic nodules were quantified. SHMT2 knockdown was found to inhibit the formation of metastatic nodules in the liver (Figure 7E). In contrast, more liver metastatic nodules were observed in mice with HT29-SHMT2^WT^ cells than in control mice (Figure 7E). The assessment of liver metastatic nodules showed that SHMT2 knockdown decreased the number of nodules by 75-80% compared with the control, while SHMT2 expression increased the nodules by 2.2-fold compared with the control (Figure 7E). These results were further confirmed by liver hematoxylin and eosin (H&E) staining (Figure 7E). Collectively, these data demonstrate that SHMT2 promotes the growth and metastasis of CRC *in vivo*.

### The SHMT2 level positively correlates with the β-catenin level in human CRC tissues and with progression and poor survival rates in patients

**Figure 8:**
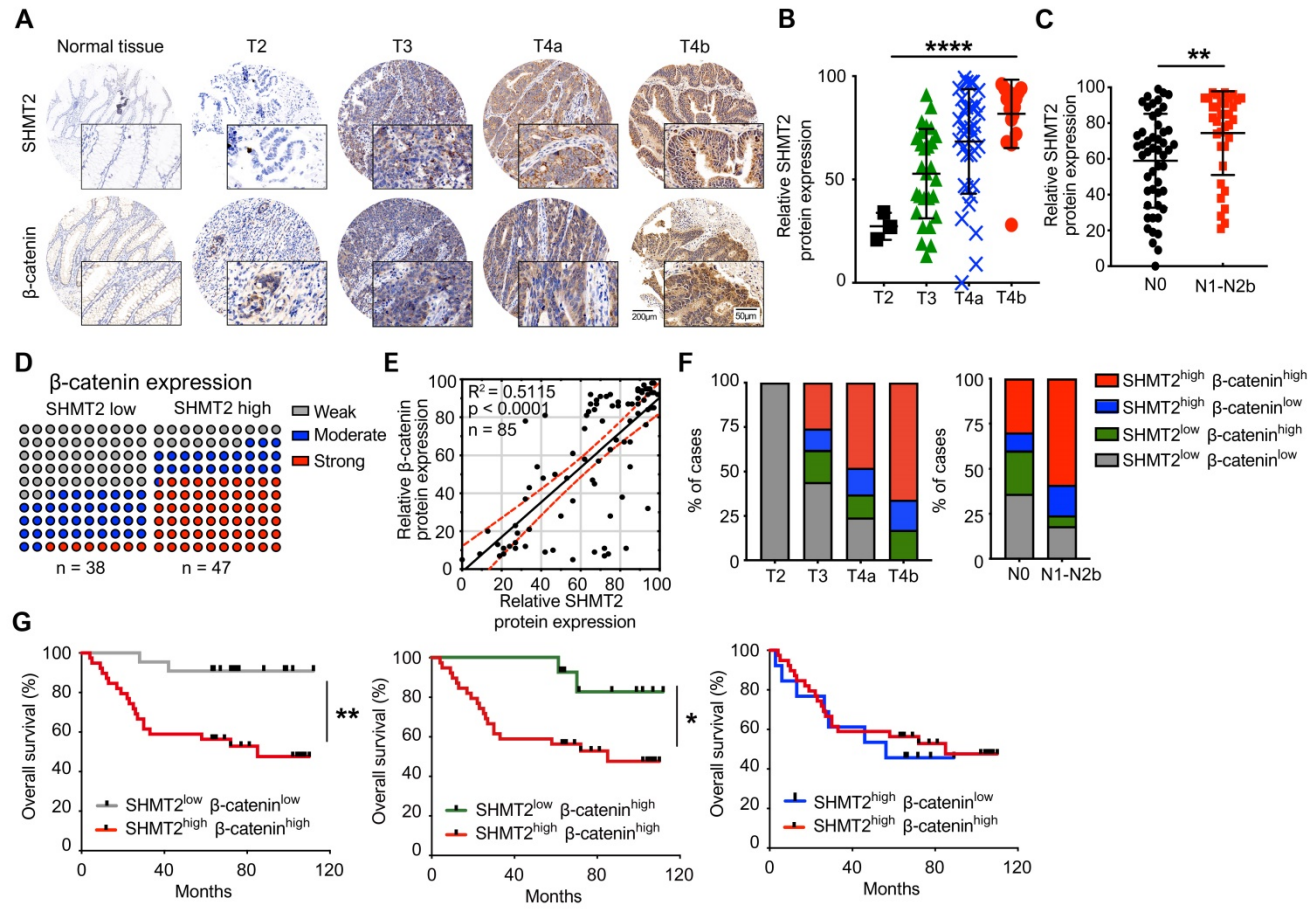
** SHMT2 level positively correlates with the level of β-catenin in human CRC tissues and the progression and poor survival rates of patients.** (A) Representative immunohistochemistry (IHC) of SHMT2 and β-catenin using human CRC tissue microarrays containing 85 human CRC specimens. Scale bar, 200 μm or 50 μm. (B) Quantification of IHC of SHMT2 based on stages of human CRC tissues. *** *p* < 0.001; ordinary one-way ANOVA test. (C) Quantification of IHC of SHMT2 in CRC tissues with or without lymph node metastasis (N0: without lymph-node metastasis; N1-N2b: with lymph node metastasis). ** *p* < 0.01; Student's t-test. (D) Quantification of β-catenin expression in human CRC tissues with low or high SHMT2 expression. (E) Pearson's test was used to analyze the correlation between SHMT2 and β-catenin protein expression in human CRC tissues. R^2^ = 0.5115; *p* < 0.0001, Pearson correlation coefficient. (F) Quantification of SHMT2 and β-catenin expression in different stages of human CRC tissues based on IHC. (G) Survival analysis was performed to analyze the relationship between patient overall survival rates and SHMT2 and β-catenin protein expression. * *p* < 0.05, ** *p* < 0.01; log-rank test.

To explore the clinical significance and correlation of SHMT2 and β-catenin in CRC, immunohistochemistry (IHC) analysis was used to validate the expression of SHMT2 and β-catenin by using a CRC tissue array that contained 85 human CRC tissues and corresponding normal adjacent tissues (NATs) (the clinical characteristics of patients are provided in Table S2). The expression of SHMT2 in CRC tissues was found to be higher than that in NATs (Figure 8A), which is consistent with Gene Expression Omnibus (GEO) data (Figure S8A). Regarding the SHMT2 IHC data, 58 cases (65%) exhibited strong immunopositivity, 18 cases (21%) exhibited moderate immunopositivity, and 12 cases (14%) exhibited no or weak immunopositivity in tumor tissues (Figure S8B). In contrast, most normal tissues (92%) exhibited no or weak SHMT2 expression (Figure S8B). The expression of SHMT2 in CRC tissues was associated with advanced T stage (*P* < 0.001) (Figure 8B) and lymph node metastasis (*P* = 0.007) (Figure 8C). β-catenin overexpression was more prominent in CRC tissues than in NATs (Figures 8A and S8B), consistent with a previous study 23. We then analyzed the potential correlation between SHMT2 and β-catenin based on the IHC data. The results showed that CRC tissues with high SHMT2 expression tended to have higher β-catenin levels, and the protein expression of SHMT2 was closely associated with that of β-catenin (R^2^ = 0.5115,* P* < 0.0001) (Figure 8D-E). Statistical analysis showed that among patients with higher SHMT2 expression, β-catenin was expressed more strongly in the nucleus (Figure S8C-D), suggesting that the upregulated β-catenin induced by SHMT2 mainly translocated into the nucleus. Furthermore, we examined the correlation between SHMT2 and β-catenin expression with cancer progression and patient survival. An analysis of SHMT2/β-catenin expression and clinicopathologic parameters showed that high SHMT2 and β-catenin expression was significantly associated with an advanced T stage and lymph node metastasis (Figure 8F). Kaplan-Meier survival analysis revealed that the expression of SHMT2 in CRC tissues was negatively correlated with the patient survival rate (Figure S8E). Notably, patients with high SHMT2 and high β-catenin expression exhibited a significantly poorer survival rate than patients with low SHMT2 and low β-catenin expression (Figure 8G). Moreover, the effect of SHMT2 on the patient survival rate was more dominant, given that the survival rate was not different between patients with high and low β-catenin expression when the SHMT2 expression was high (Figure 8G). Taken together, these results strongly suggest that the expression of SHMT2 and β-catenin is elevated in human CRC tissues and is tightly linked to CRC progression and metastasis.

## Discussion

In recent years, the nonmetabolic function of metabolic enzymes has attracted much attention. Recent studies have revealed that all essential glycolytic enzymes can be translocated into the nucleus, where they participate in tumor progression independent of their canonical metabolic roles 2, 24. These noncanonical functions include the inhibition of apoptosis, the regulation of epigenetic modifications, the modulation of transcription factors, cofactors, and extracellular cytokines which are implicated in tumorigenesis 25, 26. Here, we focused on the nonmetabolic function of SHMT2, which is a well-known enzyme responsible for glycine synthesis. We found that SHMT2 knockdown inhibited the proliferation and metastasis of CRC both *in vitro* and *in vivo*. Notably, SHMT2 is located in the cytoplasm and nucleus in addition to the mitochondria in CRC. The K64 residue of cytoplasmic SHMT2 was shown to be involved in the interaction with β-catenin, thereby suppressing the ubiquitylation degradation of β-catenin. Moreover, *SHMT2* is the target gene of β-catenin, suggesting that SHMT2 and β-catenin are involved in a positive feedback loop that enables them to mediate CRC progression and metastasis. Our findings revealed a nonmetabolic function of SHMT2 in CRC and demonstrated that SHMT2 interacts with β-catenin and inhibits its degradation.

Few studies have investigated the involvement of SHMT2 in CRC progression, and these studies mainly focused on the function of mitochondrial SHMT2 as a metabolic enzyme. They did not investigate the nonmetabolic function of SHMT2 in CRC progression 20. Based on results derived from clinical samples and the GEO database, we verified that SHMT2 is significantly upregulated in human CRC tissues and that its expression is strongly associated with a poor survival rate in patients. This is not surprising, as elevated SHMT2 expression has been observed in various human cancers, and clinically, cancer patients with high SHMT2 expression show poorer survival outcomes than those with low expression 27. We also demonstrated a positive role for SHMT2 in regulating CRC cell proliferation and metastasis, both *in vivo* and *in vitro*, suggesting that SHMT2 is a key factor that controls CRC cell growth. Interestingly, the exogenous addition of glycine and formate could not reverse the CRC cell proliferation inhibitory effect caused by SHMT2 knockdown, suggesting that SHMT2 might have other nonmetabolic biological functions that affect the proliferation of CRC cells. SHMT2 with metabolic function is known to be located in the mitochondria; however, we found that SHMT2 is also distributed in the cytoplasm and nucleus in CRC cells. This finding is consistent with previous studies. Wei et al. found that SHMT2 was located in the cytoplasm and mitochondria of HCT116 cells by western blot analysis 19. Rabl et al. found endogenous SHMT2 in the cytosol, nucleus, and mitochondria in HEK293 cells, the human colon and rectum tissues, and the human CRC cells SW620 and SW480 14. These different subcellular localizations indicate that SHMT2 may exhibit various biofunctions in addition to metabolic regulation in CRC cells. In light of these results, we sought to elucidate the nonmetabolic role of SHMT2 in CRC cells.

Through transcriptome sequencing, we found that disrupting the expression of SHMT2 caused changes in multiple signaling pathways, especially the Wnt/β-catenin pathway. More than 80% of sporadic CRC exhibit hyperactive Wnt/β-catenin signaling, which leads to β-catenin stabilization and accumulation in the cytoplasm, allowing its translocation into the nucleus and activating Wnt target genes that control cell proliferation and migration 28, 29. Our results confirmed that SHMT2 knockdown decreased the protein expression of β-catenin, whereas the mRNA expression of β-catenin did not change, prompting us to investigate β-catenin protein degradation. The most well-known degradation pathway of β-catenin is the ubiquitin (Ub)-proteasome pathway. The phosphorylation of β-catenin by CK1α and GSK-3β promotes β-catenin binding to Axin1 and β-TrCP, leading to K48 Ub-proteasome degradation of β-catenin 30. Our study showed that SHMT2 bound to β-catenin decreased the phosphorylation of β-catenin and inhibited its ubiquitin-proteasome degradation. The phosphorylation of β-catenin is a complicated process, and any modification of the above mentioned processes would affect β-catenin phosphorylation 22. For example, posttranslational modifications of Axin1, such as phosphorylation and ubiquitination, affects its stability, which in turn regulates β-catenin phosphorylation 31. We discovered that SHMT2 knockdown slightly increased the expression of Axin1 (an essential skeleton of the degradation complex) and β-TrCP (the critical E3 ligase), which may be necessary for phosphorylation inhibition of β-catenin by SHMT2, suggesting that SHMT2 may affect Axin1 or β-TrCP, thereby degrading β-catenin.

Other than the investigation of K48 Ub-proteasome degradation of β-catenin, few studies have reported that β-catenin is degraded by the autophagic lysosomal pathway 32. Petherick et al. reported that β-catenin is subject to proteasome-independent degradation via its interaction with the autophagy protein LC3 32. Panda et al. further demonstrated that AGG induces the ubiquitination of β-catenin through autophagic degradation in HT-29 cancer stem cells 33, suggesting the ubiquitin-dependent autophagic degradation of β-catenin. The status of protein ubiquitination is determined not only by the counteracting activities of E3 ubiquitin ligases but also by deubiquitinases that remove the ubiquitin moiety from the protein 34. BRCC36 is a deubiquitinase and the catalytic subunit for BRISC. SHMT2 reportedly binds to BRCC36 and acts as an apparent competitive inhibitor for BRCC36 13. Ub (K63) chains might displace bound SHMT2 from BRISC when polyubiquitylated substrates are in close proximity 13. Thus, SHMT2 is an essential mediator of BRISC association with DUB action sites. Zheng et al. found that specific binding of SHMT2 with BRISC directs BRISC activity towards K63-Ub chains conjugated to IFNAR1, allowing BRCC36 to deubiquitylate K63-Ub chains on IFNAR1/2, limiting their lysosomal degradation 12. Interestingly, we revealed that BRCC36 knockdown decreased β-catenin expression in CRC cells, suggesting that binding between SHMT2 and BRCC36 may direct BRCC36 activity towards K63-Ub chains conjugated to β-catenin in CRC cells. This observation inspired us to clarify the role of K63-Ub in β-catenin degradation. We found that SHMT2 knockdown increased the binding of β-catenin with K63-Ub, suggesting that β-catenin could be degraded by the K63-Ub pathway. This is consistent with a previous study showing that Rad6B-mediated β-catenin polyubiquitination involves ubiquitin chain extension by K63 linkages 35. Moreover, it is reported that dimeric SHMT2 is responsible for the nonmetabolic function of SHMT2 13. Walden et al., describes that it is the SHMT2 dimer binds with BRISC and direct its activity. When we treated CRC cells with PLP which decreases dimeric SHMT2, β-catenin protein level was significantly reduced (data not shown), suggesting that dimeric SHMT2 might be responsible for β-catenin stabilization which is worth further study. Collectively, cytoplasmic SHMT2 interacts with β-catenin to regulate its degradation.

We also investigated which amino acid in SHMT2 mediated its interaction with β-catenin. Previous studies suggested that the lysine residues of SHMT2 play an important role in its function. For instance, K95 acetylation of SHMT2 promotes its degradation via the TRIM21-mediated K63-ubiquitin-lysosome pathway. In this case, SHMT2-K95-Ac is enriched in the cytoplasm 19. SHMT2 desuccinylation at lysine 280 by SIRT5 activates its enzymatic activity and promotes tumor cell growth *in vitro* and *in vivo* 20. Our results showed that K64 of SHMT2 is responsible for its interaction with β-catenin, though we cannot judge from the current data whether this interaction is direct or indirect. Similar to SHMT2, previous studies show that metabolic enzymes can promote Wnt/β-catenin signaling. PKM2 facilitates its interaction with β-catenin and elicits β-catenin-induced transcriptional changes, resulting in *c-Myc* expression and cancer progression 36.

Furthermore, there is increasing evidence demonstrating that Wnt/β-catenin signaling regulates enzymes that are involved in cellular metabolism in tumors. In breast cancer, Wnt/β-catenin signaling suppresses mitochondrial respiration by reducing the transcription of the gene for cytochrome *c* oxidase and increases aerobic glycolysis 37. β-Catenin-mediated c-Myc expression results in the upregulation of several rate-limiting glycolytic genes, including those encoding glucose transporter 1, lactate dehydrogenase, and PKM2, to promote aerobic glycolysis in cancer cells 38. Moreover, some important tumor-related transcription factors, such as *ATF4, c-Myc,* and hypoxia-inducible factor-1 α (*HIF-1α*), regulate the transcription of serine metabolic pathway enzymes 39, 40. Based on previous results, we identified SHMT2 as an important direct target for the β-catenin, suggesting a positive feedback loop between SHMT2 and the Wnt/β-catenin signaling pathway which is similar with the feedback loop between Wnt/β-catenin and its target genes 41. Our study also highlights the clinical relevance and prognostic significance of SHMT2-dependent regulation of β-catenin, indicating that the assessment of SHMT2, in conjunction with β-catenin, may provide a more detailed understanding of malignant risk for individual CRC patients. In this regard, targeting SHMT2 could be a promising therapeutic strategy for CRC treatment. As yet, very few small molecular SHMT2 inhibitors that specifically inhibit the metabolic function of SHMT2 have been tested in preclinical studies 42, 43. Considering the nonmetabolic function of SHMT2, strategies that suppress the function of this protein may be more impactful. In addition to antibodies against SHMT2, proteolysis-targeting chimeric molecules (PROTACs) 44 may be a potential way to degrade SHMT2 protein. PROTACs have received considerable research attention and can hijack ubiquitin E3 ligase to ensure protein proteasome degradation, suggesting its profound potential to eliminate nonenzymatic proteins^45^, ^46^. Thus, PROTACs may couple SHMT2 inhibitors with E3 ligase β-TrCP to achieve efficient SHMT2 degradation. However, no studies have investigated the degradation of SHMT2 using PROTAC technology; such studies should be conducted in the future.

In summary, our current findings demonstrate a novel mechanism by which SHMT2 interacts with and stabilizes β-catenin, thereby promoting the transcription of β-catenin target genes and strengthening CRC cell proliferation and metastasis. The upregulation of SHMT2 in CRC is a potential indicator of aggressive CRC phenotypes and correlates with poor clinical outcomes. Our findings provide new insights into the nonmetabolic functions of metabolic enzymes and present a novel therapeutic strategy for CRC.

## Methods

### Human CRC tissue specimens

A total of 85 pairs of human CRC tissues and NAT arrays were purchased from the National Engineering Center for Biochips (Shanghai, China). Both tumor samples and NATs were histologically examined. Detailed clinicopathologic features were listed in Table S2.

### Cell lines and cell culture

Human colorectal cancer cell lines HCT116, DLD-1, HT-29, SW480, SW620, SW48, and HCT15 were obtained from American Type Culture Collection (ATCC; Manassas, VA, USA). The normal colonic epithelial cell line NCM460 was obtained from ScienCell Research Laboratories (San Diego, CA, USA). The normal colonic epithelial cell line HCoEpiC was obtained from INCELL (San Antonio, TX, USA). All cells were maintained at 37℃, and 5% CO2 in RPMI-1640 (C11875500BT, GIBCO) supplemented with 10% fetal bovine serum (FBS) (10270106, GIBCO) and penicillin-streptomycin-glutamine (100X) (10378016, GIBCO).

### siRNAs and reagents

The siRNA oligos targeting SHMT2 and BRCC36 were obtained from Ruibo (Shanghai, China). The target sequences of the siRNA oligos were as follows.

si-SHMT2 #1, 5' CCACAATCATGCCATTGCT3'

si-SHMT2 #2, 5'CCTGCAGGTTCTGAAGAAT3'

si-BRCC36 #1, 5'GCAGGAATTACAACAAGAA3'

si-BRCC36 #2, 5'GAAAGTGTGCCTTGAATCA3'

Proteasome inhibitor MG132 (S2619), Wnt pathway inhibitors XAV939 (S1180), and iCRT-14 (S8704) were purchased from Selleck (TX, USA). Cycloheximide (CHX) (239763) was purchased from Millipore (Darmstadt, Germany).

### Transfections

Cells were transfected with various siRNA oligos using Lipofectamine 2000 (11668-027, Invitrogen) according to the manufacturer's protocol. Assays were conducted 48 h post-transfection.

### RNA extraction and real-time quantitative PCR (RT-qPCR)

Total RNAs were prepared using an AxyPrep™ Multisource RNA Miniprep kit (AP-MN-MS-RNA-50, Axygen). cDNAs were prepared with a PrimeScript™ RT reagent kit with gDNA Eraser (RR047A, Takara) and processed for RT-qPCR (SsoAdvanced™ Universal SYBR^®^ Green Supermix, Bio-Rad) using the primers listed in Table S3.

### Construction of shRNA knockdown, re-expression and overexpression cell pools

SHMT2 knockdown was generated using a lentivirus-mediated delivery system, pLKD-CMV-G&PR-U6-shRNA (SHMT2) (4912, OBiO Technology, Shanghai, China). The target sequence of shRNA used in this study was shSHMT2: 5'-CCGGAGAGTTGTGGACTTT-3'. Cell pools were selected using 2 μg/ml puromycin (S7417, Selleck) for 2 weeks. The knockdown efficiency was probed by RT-qPCR or western blot analysis.

To abrogate the binding of SHMT2 shRNA, 9 silent mutations (highlighted with underline below) were introduced into the exogenous Flag-SHMT2^WT^, Flag-SHMT2α, and Flag-SHMT2^K64R^ cDNA sequences to produce an shRNA-resistant version. The shSHMT2 target sequence, CCGGAGAGTTGTGGACTTT, was converted to TAGACGGGTGGTCGATTTC. shRNA-resistant Flag-tagged SHMT2α and SHMT2^K64R^ were generated using a lentivirus-mediated delivery system, pRLenti-EF1a-mCherry-P2A-blasticidin-CMV-MCS (H12991, OBiO Technology). For the overexpression cell pools, Flag-SHMT2^WT^, Flag-SHMT2α and Flag-β-catenin were also generated using a lentivirus-mediated delivery system, pRLenti-EF1a-mCherry-P2A-blasticidin-CMV-MCS (H12991, OBiO Technology). Suspended CRC cells were infected with the prepared virus for 48 h and selected with 5 μg/ml blasticidin S HCl (S7419, Selleck) for at least 2 weeks.

### Nuclear, cytoplasmic, and mitochondrial fractionation

The nuclear, cytoplasmic, and mitochondrial fractionation were performed according to the instructions of the mitochondrial isolation kit (MP-007, Invent) and the cytoplasmic and nuclear fractionation kit (SC-003, Invent). In brief, 10-40×10^6^ cells were harvested in 250 µl of buffer A (MP-007), followed by incubation on ice for 5-10 min and vortexing vigorously for 20-30 sec. The cell suspension was transferred into a filter cartridge and centrifuged at 16,000×g for 30 sec. The pellet consisted of unbroken cells and nuclei. The supernatant was carefully transferred into a fresh 1.5 ml tube and 300 µl of buffer B (MP-007) was added, followed by centrifuging at 16,000×g for 10 min. The supernatant containing cytoplasmic protein was obtained, and the pellet was resuspended in 200 µl of buffer B (MP-007), followed by centrifuging at 8,000×g for 5 min. The supernatant was collected and 1.6 ml of cold PBS was added, followed by centrifuging at 16,000×g for 15-30 min. The mitochondria (pellet) were collected.

The unbroken cells and nuclei obtained in the above process were centrifuged at 16,000×g for 5 min. Appropriate amounts of nuclear extraction buffer (SC-003) was added to the pellet, followed by 4 rounds of vortexing vigorously for 15 sec and incubating on ice for 1 min. The nuclear extract was then immediately transferred into a pre-chilled filter cartridge with a collection tube and centrifuged at 16,000×g for 30 sec. The nuclei (pellet) were obtained.

### Cell proliferation, colony formation, and EdU incorporation assay

For the cell proliferation assay, the CRC cells were treated with siSHMT2 or siScramble. After 48 h, the cells were seeded in 96-well plates at 2 × 10^3^ cells/well. Triplicate wells were seeded for each experimental condition. In the metabolite replenishment experiment, the final concentrations of serine, glycine, and formate were 400 μM, 400 μM, and 1 mM, respectively. The corresponding metabolites were added 24 h after the cells were seeded in the 96-well plate. After various treatments, 10 μl of CCK8 solution (B34304, Bimake) was added, followed by a 2 h incubation. Cell viability was determined using a microplate reader (Thermo) at OD 450 nm every day for 4 days.

For the colony formation assay, the CRC cells were treated with siSHMT2 or siScramble. After 48 h, CRC cells (600 cells/well) were seeded into 6-well plates and incubated for approximately 14 days. The cells were fixed with 4% paraformaldehyde (PFA) and then stained with 0.5% (w/v) crystal violet. Colonies were imaged and counted.

For the EdU assay, the CRC cells were treated with siSHMT2 or siScramble. After 48 h, the cells were digested with trypsin, and cells (1-2×10^4^/well) were seeded into 96-well plates. The cell proliferation was detected using an EdU-Apollo DNA proliferation assay kit according to the manufacturer's instructions (C10810-1, RiboBio, China). Hoechst 33342 was used as a nuclear dye.

### Apoptosis analysis

For the apoptosis assay, siRNA-treated cells were stained using a BD Pharmingen™ FITC annexin V apoptosis detection kit I (556547, BD) and evaluated using a BD flow cytometer (BD, USA).

### Wound healing assay

Monolayer cells in a 6-well plate were scraped in a straight line with a 10 μl pipette tip to produce a wound. The cell was washed with PBS to remove detached cells, and incubated in a serum-free medium. Photographs of the scratch were taken at 0 h and 48 h after wounding using an OLYMPUS inverted microscope. Gap width analysis was performed with ImageJ software, and the gap width at 0 h was set to 1. Multiple defined sites along the scratch were measured, and each scratch was described as the average of all measurements. The data were presented as the average of three independent experiments.

### Migration and invasion assay

For cell migration, 1-2×10^5^ cells in 200 μl of serum-free medium were plated in an 8.0 mm, 24-well plate chamber insert (354578, Corning Life Sciences), with medium containing 10% FBS at the bottom of the insert. The cells were incubated for 24 h and then fixed with 4% PFA for 5 min. After washing 3 times with PBS, the cells were stained with 0.5% crystal violet blue for 5 min and then washed with double-distilled H_2_O. Cells on the upper surface of the insert were removed with a cotton swab. The positively stained cells were examined under the microscope. For the cell invasion assay, Matrigel-coated chambers (354483, Corning Life Sciences) were used instead of the chamber inserts used in the migration assay.

### Western blot, ubiquitin modification assay, and antibodies

Cells and tissues were lysed in RIPA buffer (P0013C, Beyotime Biotechnology) with the addition of a protease inhibitor cocktail and phosphatase inhibitor cocktail (B14001 and B15001, Bimake, USA). For the ubiquitin modification assay, the cells were lysed in lysis buffer (0.025 M Tris, 0.15 M NaCl, 0.001 M EDTA, 1% NP-40, 5% glycerol; pH 7.4). Proteins were incubated with primary antibody (1:1000) overnight at 4°C, and then incubated with HRP secondary antibody (1:10000-2000) for 1 h at room temperature (rt). The following antibodies were used: SHMT2 (12762, Cell Signaling Technology), mSHMT (sc-390641, Santa Cruz Biotechnology), ATP5A1 (A11217, ABclonal), phospho-β-catenin (Ser33/37/Thr41) (9561, Cell Signaling Technology), phospho-β-catenin (Ser45) (9564, Cell Signaling Technology), ubiquitin (3933, Cell Signaling Technology), ubiquitin (linkage-specific K48) (ab140601, Abcam), ubiquitin (linkage-specific K63) (ab179434, Abcam), β-catenin (D10A8) XP (8480, Cell Signaling Technology), β-TrCP (D13F10) (4394, Cell Signaling Technology), Axin1 (Sc-14029, Santa Cruz Biotechnology), E-cadherin (A11509, ABclonal), vimentin (10366-1-AP, Proteintech), cyclin D1 (A19038, ABclonal), Bcl-2 (ab182858, Abcam), Bax (ab182733, Abcam), cleaved caspase-3 (ab32351, Abcam), BRCC36 (A7995, ABclonal), Snail (A5243, ABclonal), Slug (A1057, ABclonal), Twist (A3237, ABclonal), ZEB1 (A5600, ABclonal), ZEB2 (A5705, ABclonal), MET (A0040, ABclonal), c-Jun (A0246, ABclonal), CD44 (A19020, ABclonal), LEF (A4473, ABclonal), MMP2 (A19080, ABclonal), MMP7 (A0695, ABclonal), MMP9 (A2095, ABclonal), β-actin (BM0627, Boster), GAPDH (A00227, Boster), lamin B1 (BM3891, Boster), Flag tag (DYKDDDDK) (D6W5B) rabbit mAb (14793, Cell Signaling Technology), AffiniPure goat anti-mouse IgG (H+L) (111-035-003, Jackson Immunoresearch), AffiniPure goat anti-rabbit IgG (H+L) (111-005-003, Jackson Immunoresearch).

### Co-immunoprecipitation (Co-IP)

Total cells were lysed in IP and western blot buffer (P0013, Beyotime Biotechnology) containing protease and phosphatase inhibitor cocktail (B14001 and B15001, Bimake). Whole lysates were incubated with 10 μg of antibodies or normal IgG. Briefly, bound proteins were eluted with 0.1 M glycine (pH 2.5) and then neutralized with 1 M Tris buffer to prevent disturbance of the heavy chain (approximately 55 kDa). The elution was analyzed by western blot.

### Immunofluorescence (IF)

CRC cells seeded on coverslips (Sarstedt Inc. TC coverslip 13 mm ST/CS200, Fisher Scientific) were fixed with 4% PFA in PBS for 20 min at rt. After washing three times with 0.2% BSA in Dulbecco's phosphate-buffered saline (DPBS), the cells were permeabilized with 0.1% Triton X-100 in PBS for 7 min. The permeabilized cells were blocked with DPBS containing 0.2% bovine serum albumin (BSA) for 30 min and then incubated with primary antibody (1:100-2000) overnight at 4°C, followed by Alexa-conjugated secondary antibody (1:200) incubation for 1 h at rt. The coverslips were mounted in DPBS supplemented with DABCO. All IF images were acquired with a laser scanning confocal microscope (Leica SP8 X, Leica) equipped with LAS X software, using a 63x3 1.3 NA oil objective. The following antibodies were used: mSHMT (sc-390641, Santa Cruz Biotechnology), TOM20 (11802-1-AP, Proteintech), β-catenin (D10A8) XP (8480, Cell Signaling Technology), E-cadherin (A11509, ABclonal), vimentin (10366-1-AP, Proteintech), goat anti-rabbit IgG (H+L) cross-adsorbed Alexa Fluor 488 (A-11008, Invitrogen), and goat anti-mouse IgG (H+L) cross-adsorbed Alexa Fluor 568 (A-11031, Invitrogen).

### Preparation of recombinant SHMT2

SHMT2α and its mutants (90-504) (ΔSHMT2) preceded by a GST purification tag and TEV cleavage sequence was cloned into a pGEX4t-1 expression vector. The constructs were expressed in *Escherichia coli* with isopropyl-β-D-thiogalactopyranoside (IPTG) induction at 37°C for 3 h. Cells were harvested by centrifugation and subsequently suspended in lysis buffer (50 mM HEPES pH 7.0, 150 mM NaCl, 0.2 mM EDTA, 1 mM DTT, 5% glycerol and 1 mM PMSF). The cell suspension was sonicated on ice, followed by centrifugation. The lysate was loaded into a GST column and eluted with 10 mM GSH in lysis buffer. All of the eluates was collected and purified by size-exclusion chromatography (Superdex 200 column; GE Healthcare) in the buffer (20 mM Tris-HCl pH 8.0, 200 mM NaCl, and 2 mM DTT). GST-SHMT2α was enriched with GST-labeled agarose beads for subsequent experiments. After TEV protease cleaved the TEV cleavage sequence, the cleaved SHMT2α and ΔSHMT2 were obtained. The protein samples were stored at -80°C until the further experiments.

### *In vitro* protein competitive binding assay

Excess and equal amounts of SHMT2α and ΔSHMT2 were added to the HCT116 cell lysates. The beads with GST-SHMT2α were added to these cell lysates and incubated overnight at 4 ℃. The beads were washed twice with PBST and boiled, and SDS-PAGE resolved the pull-down proteins for immunoblot assay.

### Chromatin immunoprecipitation (ChIP)-qPCR

ChIP was performed using the Millipore Magna ChIP G kit (17-611, Millipore) with the protocol provided. Sonicated chromatin was immunoprecipitated with either TCF4/TCF7L2 (C48H11) (2569, Cell Signaling Technology) or normal rabbit IgG (17-10109, Millipore) overnight at 4°C. RT-qPCR (SsoAdvanced™ Universal SYBR Green Supermix, Bio-Rad) was used to detect isolated ChIP fragments with the following primers.

*SHMT2*: F -5'-CCTGCCCTGAGTTTCCATTA-3',

R -5'GTCTGGTGTCGGCTCTGAT-3'.

### LC-MS analysis

CRC cells were seeded in 10 cm plates at an appropriate seeding density to be ~80% confluent by the end of the assay. The cells were harvested with 80% pre-chilled methanol and incubated at -80°C for 24 h. After centrifugation at 12,000×g for 5 min at 4°C, the supernatants were transferred to 1.5 ml glass bottles and dried by a Concentrator Plus (Eppendorf). The dried extract was derivatized by AccQ-Tag Ultra (186003836, Waters). Filtrated samples were subjected to LC-MS analysis on a UPLC-Q-TOF/MS system (Waters). For each cell, the serine and glycine abundance were normalized to the cell count.

### Mice xenografts

shNC HCT116 and shSHMT2 HCT116 or shNC DLD-1 and shSHMT2 DLD-1 cells were implanted by bilateral subcutaneous injection (1×10^7^ cells per flank) into female nude mice (Beijing HFK Bioscience, China) (n = 7-8). Vector HT-29 and SHMT2 HT-29 cells were implanted by bilateral subcutaneous injection (5×10^6^ cells per flank) into female nude mice (n = 9). The mice were monitored daily until visible, measurable tumors had formed. The tumors were measured with calipers 3 times per week. The average tumor volumes were plotted for five weeks; tumor volumes were calculated using the formula: volume = length × width^2^ /2.

All experiments adhered strictly to the limits of the project license authority (maximum permitted tumor size of 15 mm or ulceration), at which point the animals were humanely killed. In none of the experiments were these limits exceeded.

### Intrasplenic-nude mouse model system (ISMS)

CRC cells were grown to confluence and harvested as described above for subcutaneous injection. The cells were resuspended in serum-free RPMI-1640 at a density of 5×10^6^ cells/100 μl. Nude mice were anesthetized with sterile 3% pentobarbital sodium salt (50 mg/kg), and the spleen was exteriorized through a left flank incision (n = 6-8). Next, 5×10^6^ cells in 100 μl were slowly injected into the splenic pulp through a 29-gauge needle over 1 min. All animals were killed when the first mouse appeared lethargic and enlarged liver was palpated (about 6 weeks after injection). The liver tissues were analyzed by H&E staining, and liver metastasis was determined by counting the metastatic liver nodules.

### Immunohistochemistry (IHC) analysis

According to the manufacturer's instructions, paraffin-embedded tissue samples from consenting patients were incubated overnight using the following primary antibodies (1:50-100): SHMT2 (12762, Cell Signaling Technology), β-catenin (D10A8) XP (8480, Cell Signaling Technology). The IHC images were captured by a microscope (Olympus, USA). To get the relative expression (%) of the target protein, the measurement parameters collected by Image J included integral optical density (IOD), area and average optical density (AOD). The AOD represented the levels of specific protein per unit area.

### Statistical analysis

All *in vitro* experiments were performed at least three times. The results were presented as the mean ± SD. Statistical analyses were performed using GraphPad Prism and Microsoft Excel 2020. Student's t-test was used to determine significant differences in the data between two experimental groups, and one-way ANOVA was used to analyze multiple group comparisons. Pearson's correlation test was used for the correlation analysis. The log-rank test was used for the survival analysis. All statistical analyses were two-sided; * *p* < 0.05, ** *p* < 0.01 and *** *p* < 0.001 were considered significant.

## Supplementary Material

Supplementary figures and tables.
