# Supplementary material for: Cytoplasmic SHMT2 drives the progression and metastasis of colorectal cancer by inhibiting β-catenin degradation
Source: Theranostics. 2021 Jan 1;11(6):2966–86. doi: 10.7150/thno.48699 (PMC7806468; doi:10.7150/thno.48699)
Supplement: Supplementary file 1 — Supplementary figures and tables. [file thnov11p2966s1.pdf]

Supplemental Information

Cytoplasmic SHMT2 drives the progression and metastasis of colorectal cancer by inhibiting β-catenin degradation

Supporting information includes:

8 Supplementary Figures

3 Supplementary Tables

Figure S1

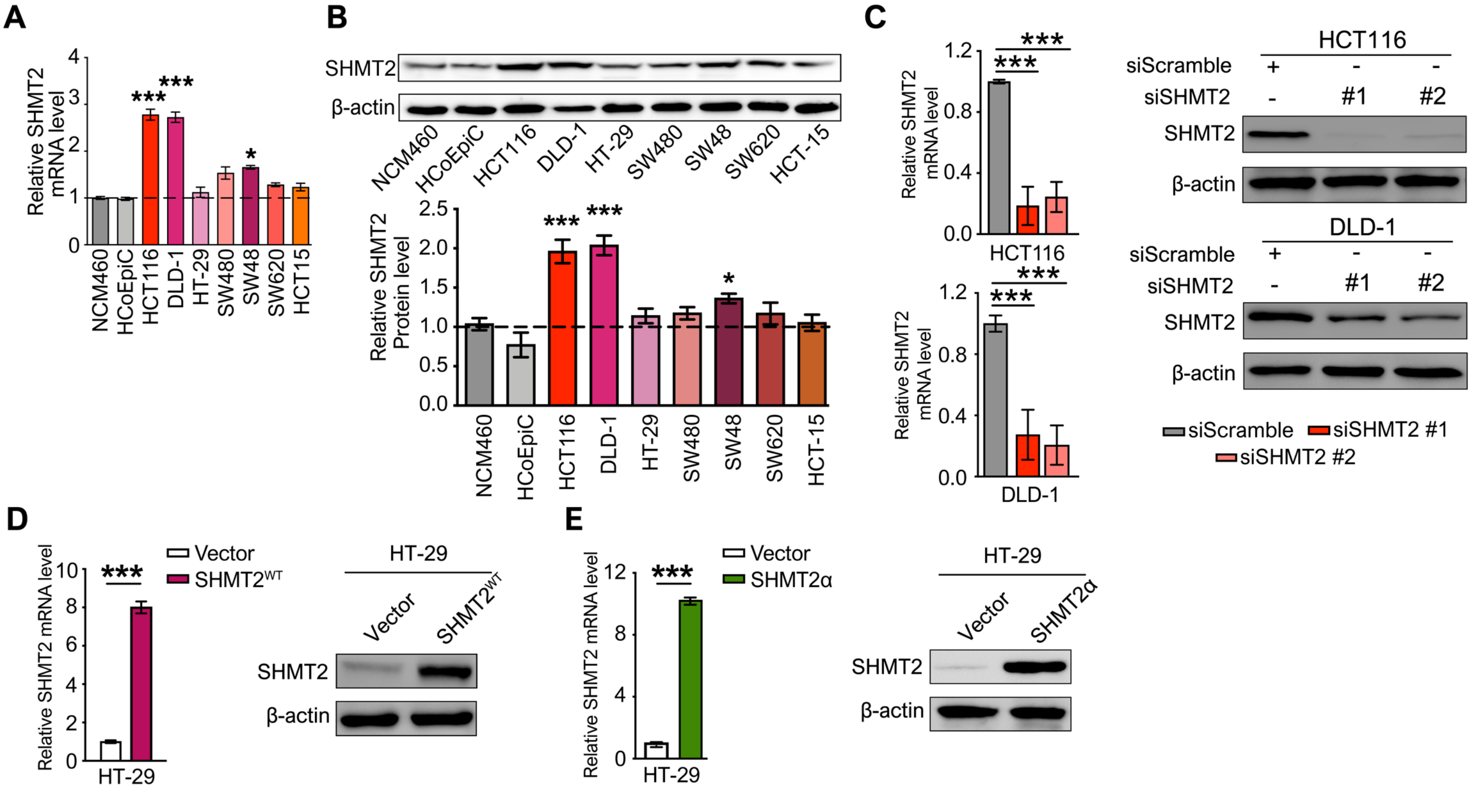
A
Relative SHMT2 mRNA level
NCM460
HCoEpiC
HCT116
DLD-1
HT-29
SW480
SW48
SW620
HCT15
B
SHMT2
β-actin
NCM460
HCoEpiC
HCT116
DLD-1
HT-29
SW480
SW48
SW620
HCT-15
Relative SHMT2 Protein level
C
Relative SHMT2 mRNA level
HCT116
DLD-1
siScramble
siSHMT2
#1
#2
SHMT2
β-actin
siScramble
siSHMT2 #1
siSHMT2 #2
D
Relative SHMT2 mRNA level
Vector
SHMT2WT
HT-29
E
Relative SHMT2 mRNA level
Vector
SHMT2α
HT-29

Figure S2

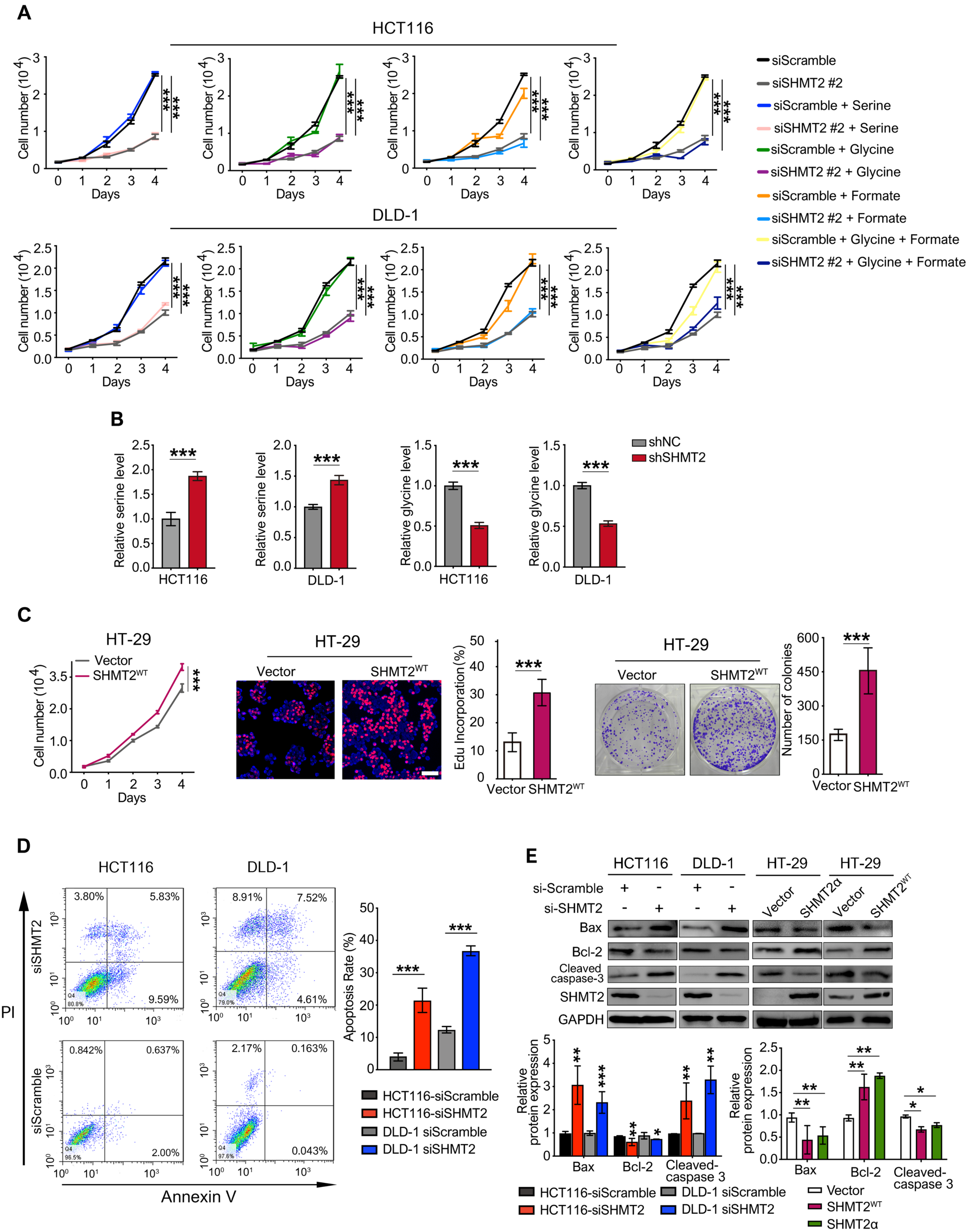

A
HCT116
DLD-1
Cell number (10^4)
Days
siScramble
siSHMT2 #2
siScramble + Serine
siSHMT2 #2 + Serine
siScramble + Glycine
siSHMT2 #2 + Glycine
siScramble + Formate
siSHMT2 #2 + Formate
siScramble + Glycine + Formate
siSHMT2 #2 + Glycine + Formate
B
Relative serine level
Relative glycine level
shNC
shSHMT2
HCT116
DLD-1
C
HT-29
Vector
SHMT2WT
Edu Incorporation (%)
Number of colonies
D
HCT116
DLD-1
siSHMT2
siScramble
PI
Annexin V
3.80%
5.83%
9.59%
8.91%
7.52%
4.61%
0.842%
0.637%
2.00%
2.17%
0.163%
0.043%
Apoptosis Rate (%)
HCT116-siScramble
HCT116-siSHMT2
DLD-1 siScramble
DLD-1 siSHMT2
E
si-Scramble
si-SHMT2
SHMT2α
Bax
Bcl-2
Cleaved caspase-3
SHMT2
GAPDH
Relative protein expression
Cleaved-caspase 3

Figure S3

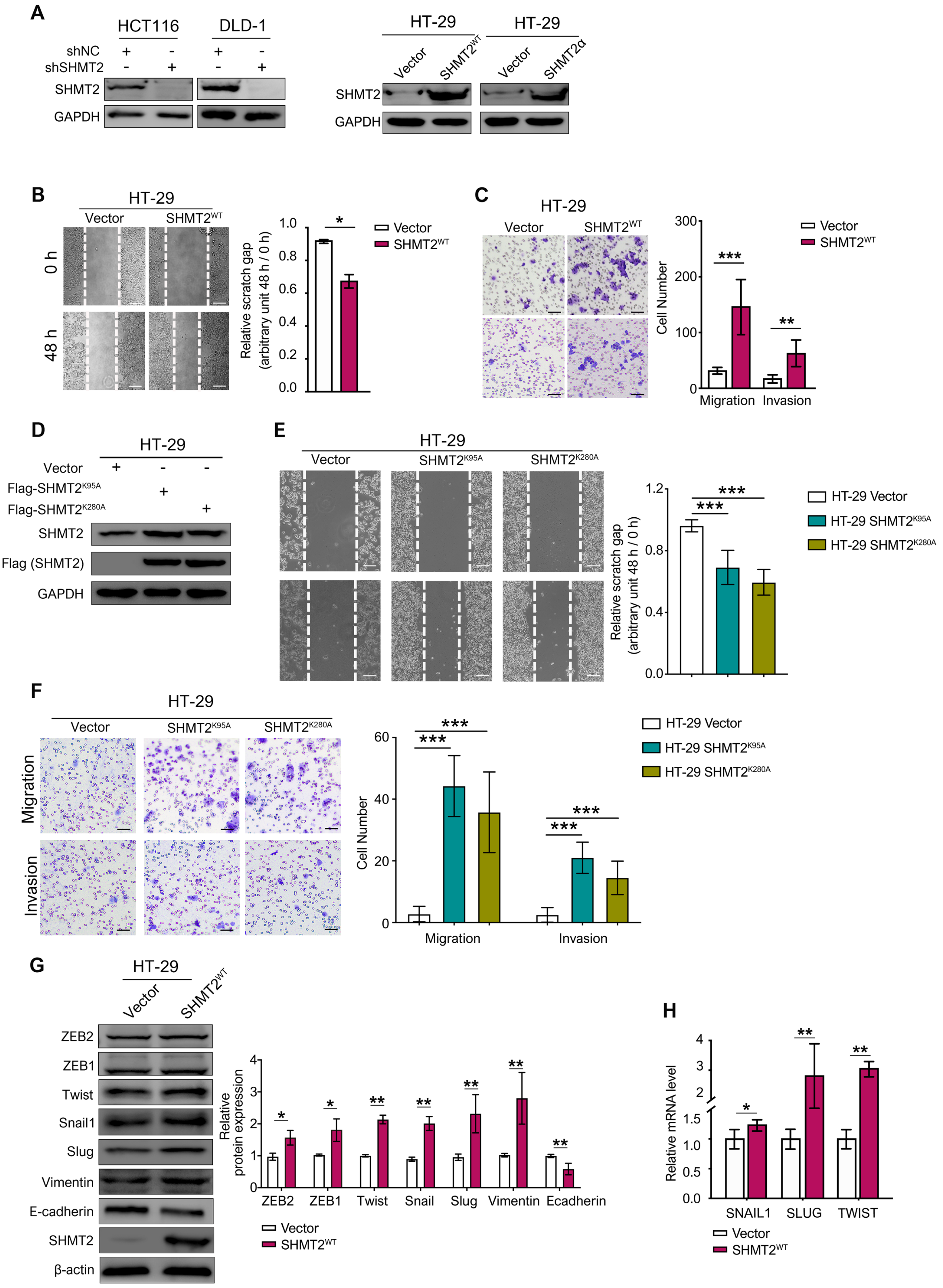

A
HCT116
DLD-1
shNC
shSHMT2
SHMT2
GAPDH
HT-29
Vector
SHMT2WT
SHMT2α
B
HT-29
Vector
SHMT2WT
0 h
48 h
Relative scratch gap (arbitrary unit 48 h / 0 h)
C
Cell Number
Migration
Invasion
D
Flag-SHMT2K95A
Flag-SHMT2K280A
Flag (SHMT2)
E
SHMT2K95A
SHMT2K280A
HT-29 Vector
HT-29 SHMT2K95A
HT-29 SHMT2K280A
F
G
ZEB2
ZEB1
Twist
Snail1
Slug
Vimentin
E-cadherin
β-actin
Relative protein expression
Snail
Ecadherin
H
Relative mRNA level
SNAIL1
SLUG
TWIST

Figure S4

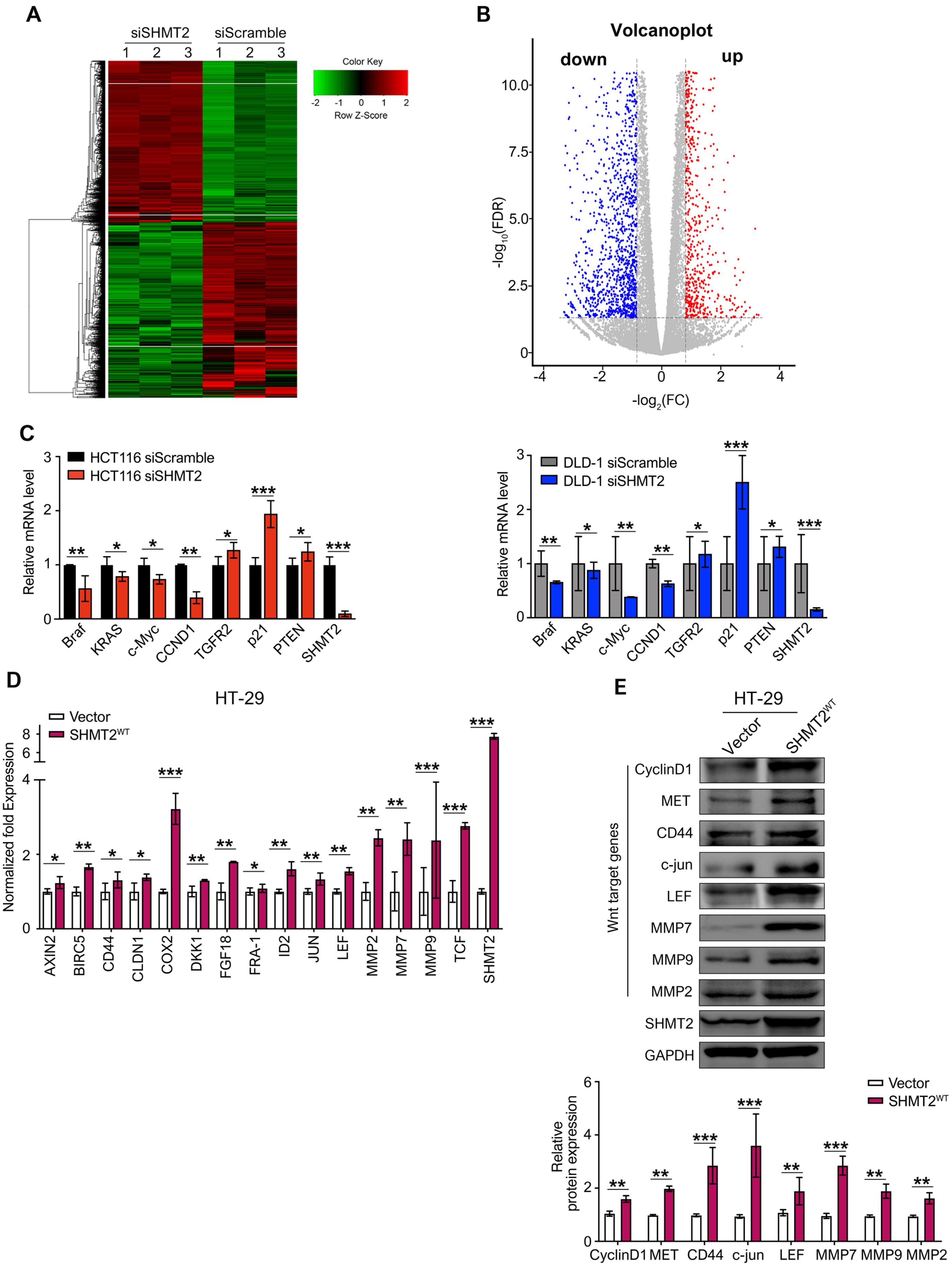
A
siSHMT2
siScramble
1 2 3 1 2 3
Color Key
-2 -1 0 1 2
Row Z-Score
B
Volcanoplot
down
up
$-\log_{10}(FDR)$
$-\log_2(FC)$
C
HCT116 siScramble
HCT116 siSHMT2
DLD-1 siScramble
DLD-1 siSHMT2
Relative mRNA level
Braf KRAS c-Myc CCND1 TGFR2 p21 PTEN SHMT2
D
HT-29
Vector
SHMT2WT
Normalized fold Expression
AXIN2 BIRC5 CD44 CLDN1 COX2 DKK1 FGF18 FRA-1 ID2 JUN LEF MMP2 MMP7 MMP9 TCF SHMT2
E
HT-29
Vector
SHMT2WT
Wnt target genes
CyclinD1
MET
CD44
c-jun
LEF
MMP7
MMP9
MMP2
SHMT2
GAPDH
Relative protein expression
CyclinD1 MET CD44 c-jun LEF MMP7 MMP9 MMP2

Figure S5

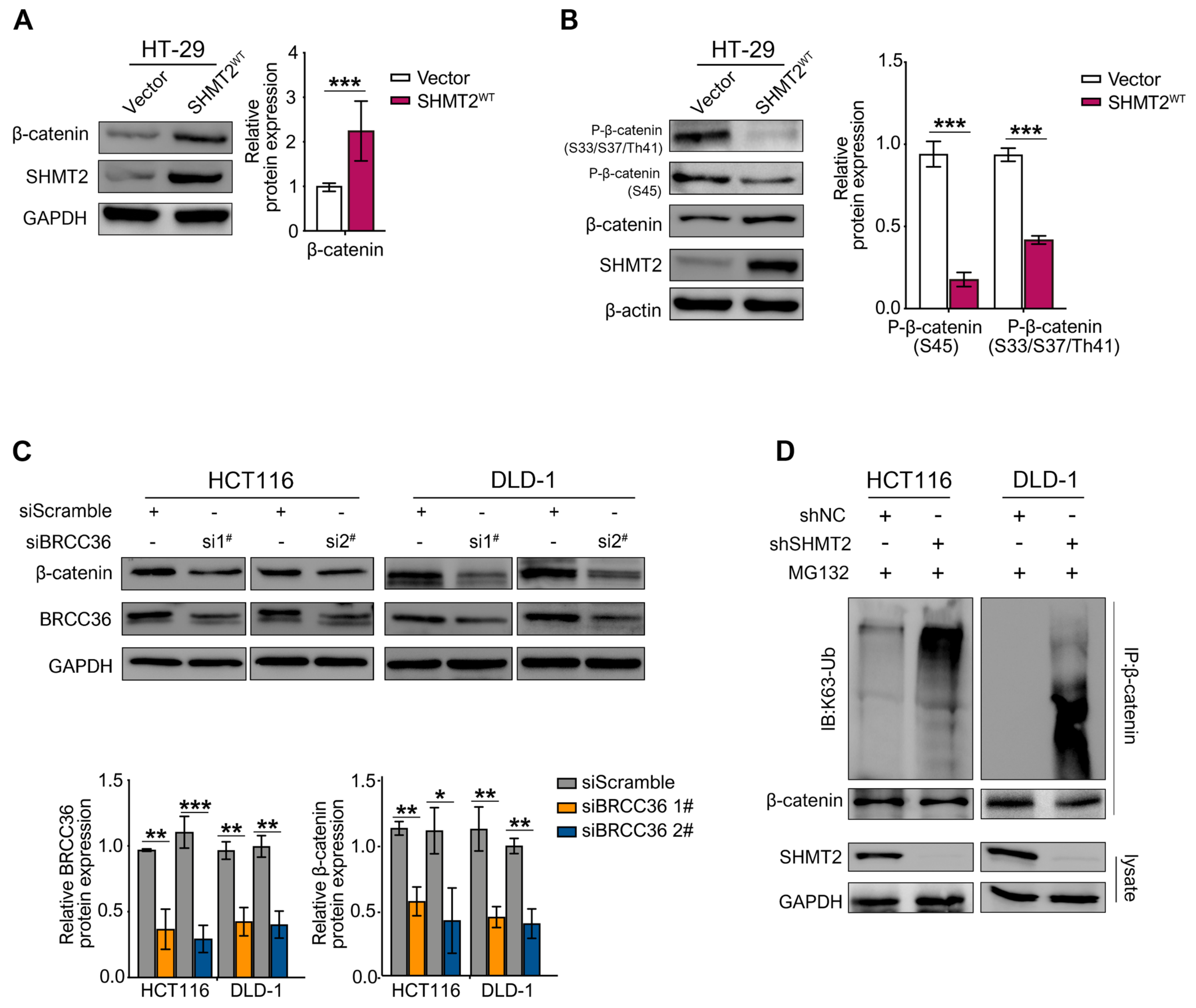

A
HT-29
Vector
SHMT2WT
β-catenin
SHMT2
GAPDH
Relative protein expression
***
Vector
SHMT2WT
β-catenin
B
HT-29
Vector
SHMT2WT
P-β-catenin (S33/S37/Th41)
P-β-catenin (S45)
β-catenin
SHMT2
β-actin
Relative protein expression
***
***
Vector
SHMT2WT
P-β-catenin (S45)
P-β-catenin (S33/S37/Th41)
C
HCT116
DLD-1
siScramble
siBRCC36
si1#
si2#
β-catenin
BRCC36
GAPDH
Relative BRCC36 protein expression
Relative β-catenin protein expression
HCT116
DLD-1
siScramble
siBRCC36 1#
siBRCC36 2#
D
HCT116
DLD-1
shNC
shSHMT2
MG132
IB:K63-Ub
β-catenin
IP:β-catenin
SHMT2
GAPDH
lysate

Figure S6

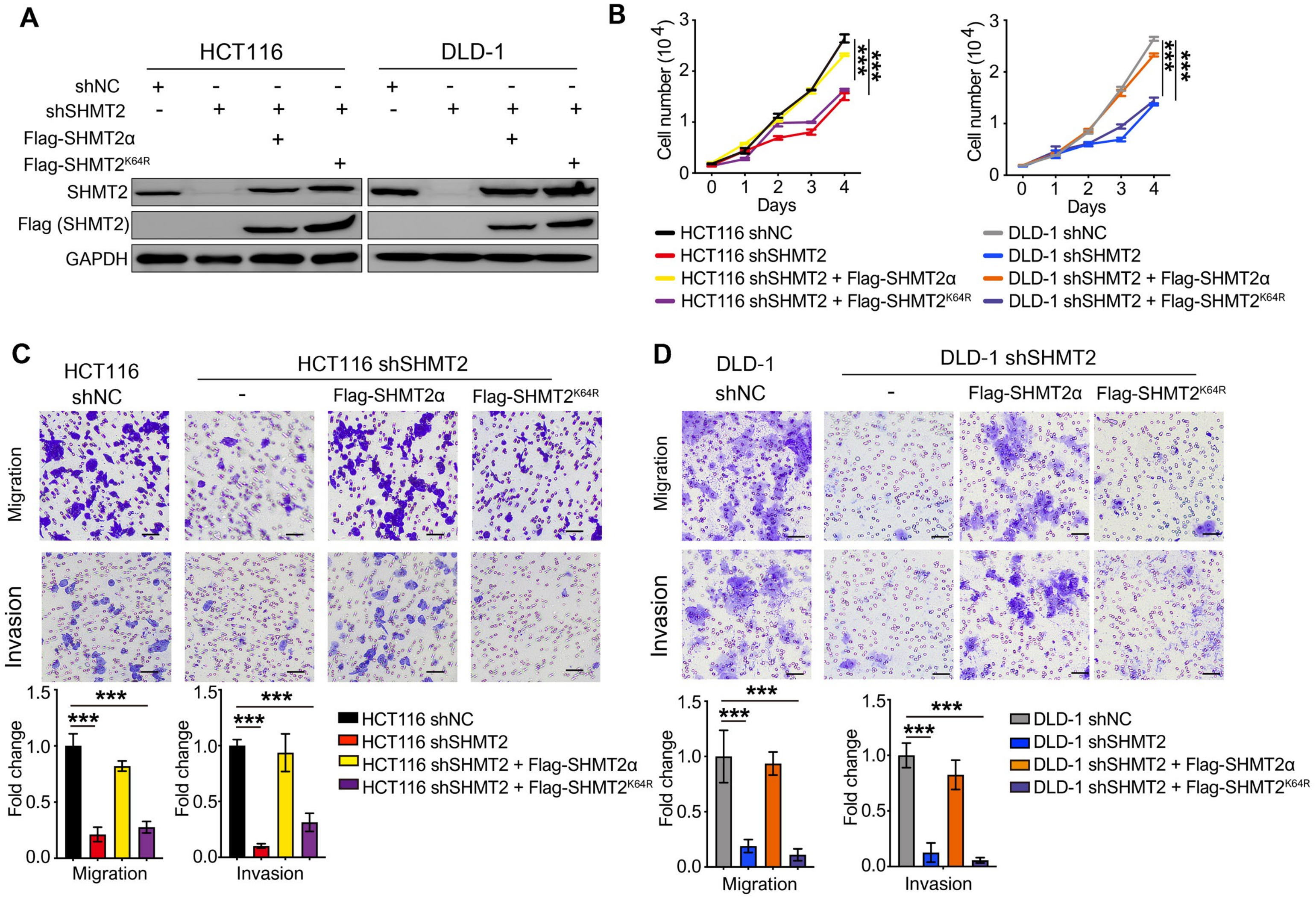

A
HCT116
DLD-1
shNC
shSHMT2
Flag-SHMT2α
Flag-SHMT2K64R
SHMT2
Flag (SHMT2)
GAPDH
B
Cell number (10^4)
Days
HCT116 shNC
HCT116 shSHMT2
HCT116 shSHMT2 + Flag-SHMT2α
HCT116 shSHMT2 + Flag-SHMT2K64R
DLD-1 shNC
DLD-1 shSHMT2
DLD-1 shSHMT2 + Flag-SHMT2α
DLD-1 shSHMT2 + Flag-SHMT2K64R
C
HCT116 shNC
HCT116 shSHMT2
Flag-SHMT2α
Flag-SHMT2K64R
Migration
Invasion
Fold change
D
DLD-1 shNC
DLD-1 shSHMT2

Figure S7

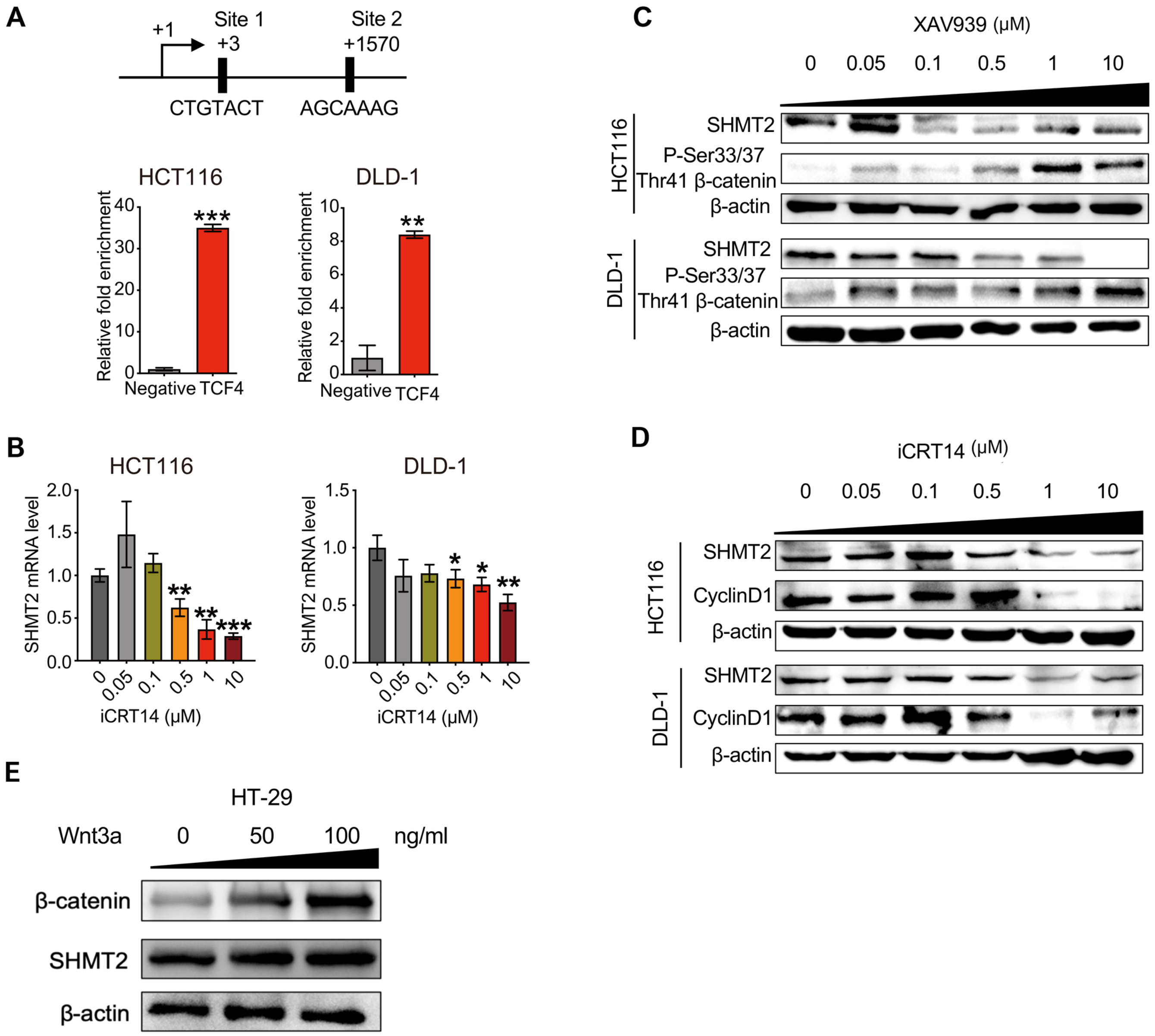

A
+1
Site 1
+3
Site 2
+1570
CTGTACT
AGCAAAG
HCT116
DLD-1
Relative fold enrichment
Negative
TCF4
***
**
B
HCT116
DLD-1
SHMT2 mRNA level
iCRT14 (μM)
0
0.05
0.1
0.5
1
10
C
XAV939 (μM)
SHMT2
P-Ser33/37
Thr41 β-catenin
β-actin
HCT116
DLD-1
D
iCRT14 (μM)
CyclinD1
E
HT-29
Wnt3a
0
50
100
ng/ml
β-catenin

Figure S8

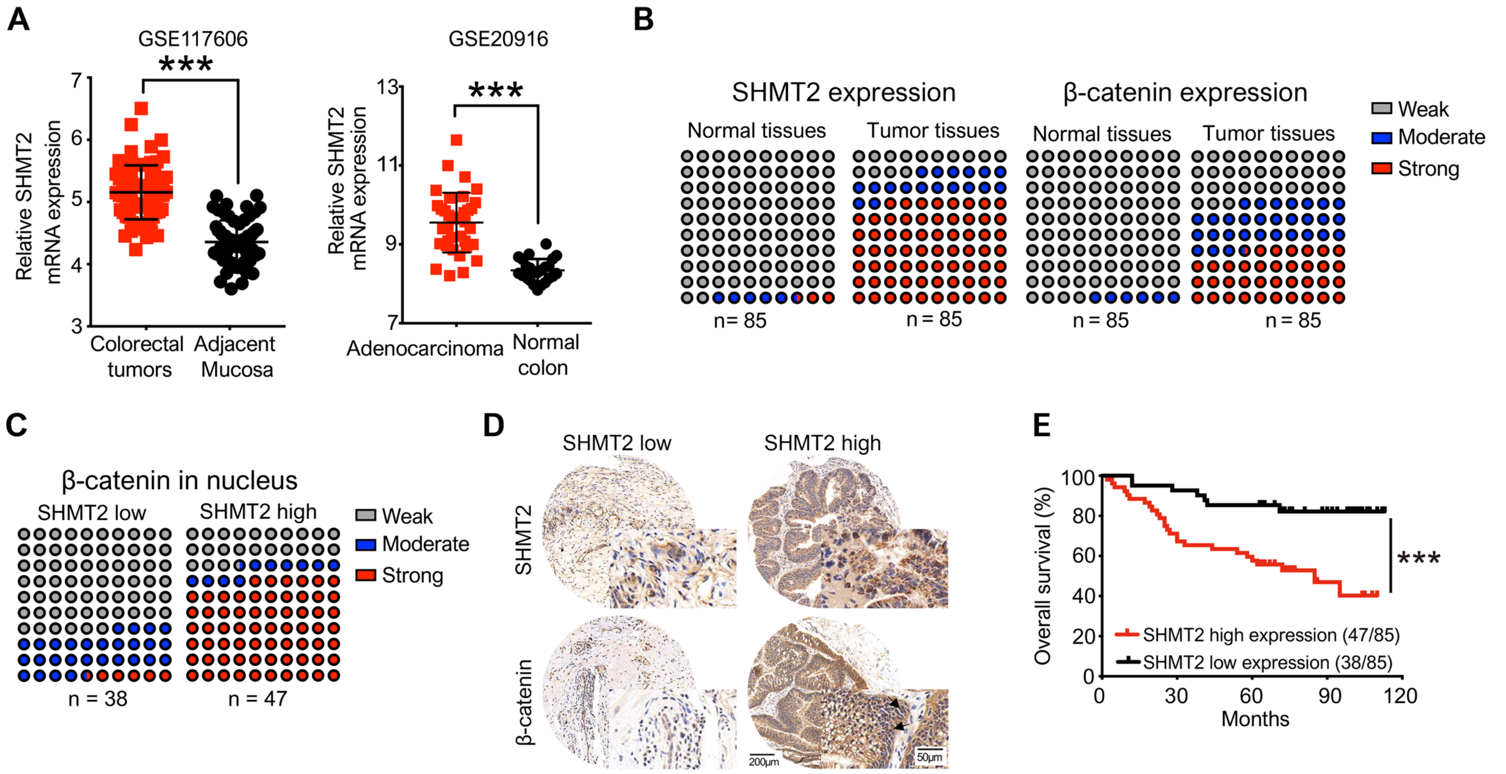

A
GSE117606
Relative SHMT2 mRNA expression
Colorectal tumors
Adjacent Mucosa
***
GSE20916
Relative SHMT2 mRNA expression
Adenocarcinoma
Normal colon
***
B
SHMT2 expression
β-catenin expression
Normal tissues
Tumor tissues
Normal tissues
Tumor tissues
n= 85
n = 85
n = 85
n = 85
Weak
Moderate
Strong
C
β-catenin in nucleus
SHMT2 low
SHMT2 high
n = 38
n = 47
Weak
Moderate
Strong
D
SHMT2 low
SHMT2 high
SHMT2
β-catenin
200μm
50μm
E
Overall survival (%)
Months
SHMT2 high expression (47/85)
SHMT2 low expression (38/85)
***

Figure S1. SHMT2 is overexpressed in human CRC cells. Related to Figure 1.

(A) The relative mRNA level of SHMT2 in human CRC cells and normal colonic epithelial cell NCM460 and HCoEpiC. * $p < 0.05$, *** $p < 0.001$; Student's t-test.

(B) Western blot analysis (up) and quantification (bottom) of SHMT2 in human CRC cells and normal colonic epithelial cell NCM460 and HCoEpiC. * $p < 0.05$, *** $p < 0.001$; Ordinary one-way ANOVA multiple comparisons test.

(C) The knockdown of SHMT2 by siRNA in HCT116 and DLD-1 cells were confirmed by RT-qPCR and western blot analysis. *** $p < 0.001$; Ordinary one-way ANOVA multiple comparisons test.

(D) (E) The overexpression of $SHMT2^{WT}$ and SHMT2α in HT-29 cells was confirmed by RT-qPCR and western blot analysis. *** $p < 0.001$; Student's t-test.

Data are presented as the mean ± SD from at least three independent experiments. The error bars show the SD.

Figure S2. SHMT2 promotes CRC cell proliferation and regulates cell apoptosis. Related to Figure 1.

(A) Cells proliferation wase detected by CCK8 assay by siSHMT2 #2, with or without 400 μM serine, 400 μM glycine or 1mM formate supplementation (n = 3). *** $p < 0.001$; Ordinary one-way ANOVA multiple comparisons test.

(B) CRC cells with shSHMT2 were harvested in 80% confluence. Serine and glycine levels in cells were analyzed by LC-MS (n = 3). The relative quantities of metabolites were shown. *** $p < 0.001$; Student's t-test.

(C) Representative images and quantification of cell proliferation (left) (n = 3), EdU incorporation assay (middle) (n = 5) and colony formation assay (right) (n = 3) using HT29 cells with $SHMT2^{WT}$ overexpression. Scale bar, 50 μm. ** $p < 0.01$; Student's t-test.

(D) HCT116 and DLD-1 cells with siSHMT2 were stained by annexin V/propidium iodide (PI), followed by flow cytometry (n = 3). The relative quantities of the apoptosis rate (%) were shown. *** $p < 0.001$; Student's t-test.

(E) Western blot analysis (up) and quantification (bottom) of SHMT2, BAX, Bcl-2 and Cleaved-caspase-3 in CRC cells with SHMT2 knockdown, SHMT2α and $SHMT2^{WT}$ overexpression. * $p < 0.1$, ** $p < 0.05$, *** $p < 0.001$; Student's t-test and Ordinary one-way ANOVA multiple comparisons test.

Data are presented as the mean ± SD from at least three independent experiments. The error bars show the SD.

Figure S3. The catalytic function of SHMT2 is not indispensable for the migration and invasion ability of HT-29 cells. Related to Figure 2.

(A) The knockdown of SHMT2 by shRNA in HCT116 and DLD-1 cells were confirmed by western blot analysis. The overexpression of $SHMT2^{WT}$ and SHMT2α in HT-29 cells was confirmed by western blot analysis.

(B) Representative images (left) and quantification (right) of the wound healing assay using HT-29 cells with $SHMT2^{WT}$ overexpression (n = 3). Scale bar, 200 μm. * $p < 0.05$; Student's t-test.

(C) Representative images (left) and quantification (right) of the migration and invasion assay using HT-29 cells with $SHMT2^{WT}$ overexpression (n = 3). Scale bar, 200 μm. ** $p < 0.01$; Student's t-test.

(D) The overexpression of Flag-$SHMT2^{K95A}$ and Flag-$SHMT2^{K280A}$ in HT-29 cells were confirmed by western blot analysis.

(E) Representative images (left) and quantification (right) of the wound healing assay using HT-29 cells with Flag-$SHMT2^{K95A}$ and Flag-$SHMT2^{K280A}$ overexpression (n = 3). Scale bar, 200 μm. *** $p < 0.001$; Ordinary one-way ANOVA multiple comparisons test.

(F) Representative images (left) and quantification (right) of migration and invasion assay using HT-29 cells with Flag-SHMT2^{K95A} and Flag-SHMT2^{K280A} overexpression (n = 3). Scale bar, 100 μm. *** $p < 0.001$; Ordinary one-way ANOVA multiple comparisons test.

(G) Western blot analysis (left) and quantification (right) of vimentin, E-cadherin and EMT-associated transcription factors in HT-29 cells after SHMT2^{WT} overexpression. * $p < 0.05$, ** $p < 0.01$; Student's t-test.

(H) RT-qPCR analysis of EMT-associated transcription factors in HT-29 cells after SHMT2^{WT} overexpression (n = 3). * $p < 0.05$, ** $p < 0.01$; Student's t-test.

Data are presented as the mean ± SD from at least three independent experiments. The error bars show the SD.

Figure S4. SHMT2 knockdown affects the expression of multiple genes in CRC cells. Related to Figure 3.

(A) Heatmap and sample clustering analysis of the mRNA-seq analysis of HCT116 cells after SHMT2 knockdown. Each column represents a sample, and each row represents a gene. Red represents upregulated genes, and the green represents downregulated genes (n = 3).

(B) Volcano plots of the mRNA-seq analysis of HCT116 cells after SHMT2 knockdown. Blue dots represent downregulated genes, and red dots represent upregulated genes (FDR < 0.05) (n = 3).

(C) RT–qPCR was performed to verify the representative genes obtained from mRNA-seq results in HCT116 and DLD-1 cells (n = 3). * $P < 0.05$, ** $P < 0.01$, *** $P < 0.001$; Student's t-test.

(D) RT-qPCR analysis of Wnt target genes with SHMT2^{WT} overexpression (n = 3). * $p < 0.05$, ** $p < 0.01$, *** $p < 0.001$; Student's t-test.

(E) Western Blot analysis (top) and quantification (bottom) of Wnt target genes in HT-29 cells with SHMT2^{WT} overexpression. * $p < 0.05$, ** $p < 0.01$, *** $p < 0.001$;

Student's t-test.

Data are presented as the mean ± SD from at least three independent experiments. The error bars show the SD.

Figure S5. BRCC36 knockdown inhibits the β-catenin expression, and SHMT2 knockdown increases K63-Ub of β-catenin. Related to Figure 4.

(A) Western blot analysis (left) and quantification (right) of β-catenin in HT-29 cells after $SHMT2^{WT}$ overexpression. *** $p < 0.001$; Student's t-test.

(B) Western blot analysis (left) and quantification (right) of phosphorylated β-catenin after $SHMT2^{WT}$ overexpression in HT-29 cells. *** $p < 0.001$; Student's t-test.

(C) Western blot analysis (up) and quantification (bottom) of β-catenin after BRCC36 knockdown in CRC cells. * $p < 0.1$, ** $p < 0.05$, *** $p < 0.001$; Student's t-test.

(D) CRC cells stably expressing shNC or shSHMT2 were treated with MG132 for 16 h before harvesting. β-catenin was immunoprecipitated and immunoblotted with the indicated antibodies.

Data are presented as the mean ± SD from at least three independent experiments. The error bars show the SD.

Figure S6. K64 of SHMT2 is critical for CRC cell proliferation, migration, and invasion. Related to Figure 5.

(A) The overexpression of Flag-SHMT2α and Flag-$SHMT2^{K64R}$ in HCT116 and DLD-1 was confirmed by western blot analysis.

(B) Cell proliferation was detected by a CCK8 assay after stable SHMT2 knockdown with the re-expression of shRNA-resistant SHMT2α or $SHMT^{K64R}$ (n = 3). *** $p < 0.001$; Ordinary one-way ANOVA multiple comparisons test.

(C-D) Representative images (top) and statistically analyzed (bottom) of the transwell migration and invasion assay using CRC cells after stable SHMT2 knockdown with the

re-expression of shRNA-resistant SHMT2α or SHMT^{K64R} (n = 3). Scale bar, 100 μm. *** $p < 0.001$; Ordinary one-way ANOVA multiple comparisons test.

Data are presented as the mean ± SD from at least three independent experiments. The error bars show the SD.

Figure S7. SHMT2 is a target gene of the Wnt/β-catenin pathway

(A) The software PROMO was used to identify a putative binding site of TCF4 (TCF4 BS) in the SHMT2 gene (top). ChIP-qPCR analysis of the enrichment of TCF-4 on the SHMT2 promoter in HCT116 and DLD-1 cells (bottom) (n = 3). ** $p < 0.01$, *** $p < 0.001$; Student's t-test.

(B) RT-qPCR analysis of SHMT2 expression in HCT116 and DLD-1 cells treated with Wnt signal inhibitors iCRT14 (n = 3). * $p < 0.05$, ** $p < 0.01$, *** $p < 0.001$; Ordinary one-way ANOVA multiple comparisons test.

(C) Western blot analysis of SHMT2 and phosphorylated β-catenin expression in HCT116 and DLD-1 cells treated with the Wnt signal inhibitor XAV939.

(D) Western blot analysis of SHMT2 and Cyclin D1 expression in HCT116 and DLD-1 cell treated with the Wnt signal inhibitor iCRT14.

(E) Western blot analysis of SHMT2 and β-catenin in HT-29 cells with Wnt3a treatment.

Data are presented as the mean ± SD from at least three independent experiments. The error bars show the SD.

Figure S8. SHMT2 and β-catenin expression in CRC patients. Related to Figure 8.

(A) The relative mRNA expression of SHMT2 in two GEO datasets (GSE117606 and GSE20916). Data are presented as the mean ± SD. The error bars show the SD. ***$P < 0.001$; Student's t-test.

(B) Quantitation of SHMT2 and β-catenin expression in CRC tissues and NATs in 85 human CRC specimens.

(C) The expression of nucleus β-catenin in CRC tissues with low or high expression of

SHMT2.

(D) Representative IHC staining images of SHMT2 and β-catenin in human CRC tissues. The arrow points to the position where β-catenin enters the nucleus: scale bar, 200 μm, or 50 μm.

(E) Survival analysis of the correction of SHMT2 expression in CRC tissues with patients' survival rate. ***$P < 0.001$; Log-rank test.

Table S1. Go pathway analysis. Related to Figure 3.

Style	Pathway Term	DifGene	P-Value	Enrichment
down	Wnt signaling pathway	15	0.0233	1.3669
down	TGF-β signaling pathway	12	0.0190	1.9454
down	Arginine and proline metabolism	8	0.0281	1.8905
down	Alanine, aspartate and glutamate metabolism	6	0.0308	2.3237
down	Glycine, serine, and threonine metabolism	6	0.0272	2.1449
down	Notch signaling pathway	6	0.0326	1.7428
down	beta-Alanine metabolism	5	0.0298	2.3237
down	Histidine metabolism	5	0.0373	2.2487

Table S2. Clinicopathologic characters of CRC patients. Related to Figure 8.

Characteristic	Cohort 1, n
Patients	85
Age, yr (median, range)	63 (33-86)
Gender	
Male	49 (57%)
Female	36 (43%)
T stages	
T1-T2	3 (3%)
T3-T4	82 (97%)
Lymph node involvement	
No	51 (60%)
Yes	34 (40%)
Distance metastasis	
No	85 (100%)
Yes	N
AJCC stages	
I-II	49 (57%)
III-IV	36 (43%)

Table S3. Primers used in this study.

Primer set	Forward	Reverse
β-actin	TGACGTGGACATCCGCAAAG	CTGGAAGGTGGACAGCGAGG
SHMT2	CCTGCCCTGAGTTTCCATTA	GTCTGGTGTCGGCTCTGAT
TGFR2	CGGTTAA TAACGACATGATAGTCAC	TCATGGCAAACTGTCTCTAGTGTTA
p21	GGACAGCAGAGGAAGACCATGT	TGGAGTGGTA GAAATCTGTCATGC
c-Myc	GCCACGTCTCCACACATCAG	TGGTGCATTTTCGGTTGTTG
PTEN	CAATGTTCAGTGGCGGAA	TCGTGTGGGTCCTGAATT
KRAS	ACAGAGAGTGGAGGATGCTTT	TTTCACACAGCCAGGAGTCTT
BRAF	CAAACTTATAGATATTGCACA	TCTGGTGCCATCCACAAAATG
CCND1	CCCTCGGTGTCCTACTTCA	CTCCTCGCACTTCTGTTCCT
TCF	CTCCGATGTCCACTTTCCAT	CGCTTCCTCTATTTGCCATT
CTNNB1	TGGTGACAGGGAAGACATCA	CCATAGTGAAGGCGAACTGC
CD44	TCAGAGGAGTCGGAGAGAGGAAAC	GAAAAGTCAAAGTAACAATAACGTGG
COX2	CGGCGGACTAATCTTCAACT	CGGTGAAAGTGGTTTGGTTT
FGF18	AGTGGGAAACACATCCAGGT	CGGACTTGACTACCGAAGG
FRA-1	TGACCACACCCTCCCTAACT	CTGCTGCTACTCTTGCGATG
c-JUN	AAAGGAAGCTGGAGAGAATCG	TGTTCCCTGAGCATGTTGG
MET	TGCACAGTTGGTCCTGCCATGA	CAGCCATAGGACCGTATTTCGG
LEF	ACCTCTTGGCTGGTTTTCCGTCA	GTCAGTGTGGGGATGTTCCT
MMP2	CCATCGAGACCATGCGGAAGC	CATCGCTCCAGACTTGGAAGGC
MMP7	ACCGTGCTGTGTGCTGTGT	GTCCTGAGCCTGTTCCCACT
MMP9	TTCTGCCAGGACCGCTTCTAC	AGGATGTCATAGGTCACGTAGCC
Twist	CGGACAAGCTGAGCAAGATT	ACGGAGAAGGCGTAGCTGA
Slug	CAGCTCCACTCCACTCTCCT	TGAACCACTGTGATCCTTGG
Snail	CACACTGGTGAGAAGCCATT	GCCTGGCACTGGTATCTCTT
